# pastboon: an R package to simulate parameterized stochastic Boolean networks

**DOI:** 10.1093/bioadv/vbaf017

**Published:** 2025-02-06

**Authors:** Mohammad Taheri-Ledari, Sayed-Amir Marashi, Kaveh Kavousi

**Affiliations:** Laboratory of Complex Biological Systems and Bioinformatics (CBB), Department of Bioinformatics, Institute of Biochemistry and Biophysics (IBB), University of Tehran, Tehran, 1417614411, Iran; Department of Biotechnology, College of Science, University of Tehran, Tehran, 1417614411, Iran; Laboratory of Complex Biological Systems and Bioinformatics (CBB), Department of Bioinformatics, Institute of Biochemistry and Biophysics (IBB), University of Tehran, Tehran, 1417614411, Iran

## Abstract

**Summary:**

Influencing the behavior of a Boolean network involves applying perturbations, which, in standard deterministic Boolean networks, is equivalent to modifying the update rules. Nevertheless, manipulating update functions to make a Boolean network exhibit the desired dynamics is challenging, as it requires extensive knowledge of the rationale behind the logical equations. Moreover, modifying logical rules can inadvertently alter essential functional and behavioral characteristics of the network. An alternative approach is to incorporate a set of parameters into the logical functions of Boolean networks. With such methods, one can alter the behavior of the network without needing detailed knowledge of the logical functions. We developed pastboon, an R package to simulate parameterized stochastic Boolean networks using three parameterization methods. This package enables researchers to study the phenotypic effects of various perturbations on Boolean network models describing cellular processes, which find valuable applications in systems biology.

**Availability and implementation:**

pastboon is freely available on the R CRAN repository at https://cran.r-project.org/package=pastboon, and its source code can be accessed on GitHub at https://github.com/taherimo/pastboon.

## 1 Introduction

Boolean networks (BNs) are mathematical models representing complex systems as a collection of binary variables connected through Boolean logic interactions, called update rules. BN modeling plays an important role in the study of complex biological systems, capturing essential interactions and dynamics. BN models have valuable applications in various biological domains, providing critical insights into cellular processes such as gene regulation and signal transduction (Kholodenko *et al.* 2012, Radde and Hütt 2016).

Influencing the behavior of a BN involves applying perturbations, which, in standard deterministic BNs, is equivalent to modifying the update rules. Nevertheless, manipulating update functions to make the BN exhibit desired dynamics is challenging, as it requires extensive knowledge of the rationale behind the logical equations. Moreover, modifying logical rules can inadvertently alter essential functional and behavioral characteristics of the network. An alternative approach to modifying a BN’s behavior is parameterization, which involves incorporating a set of parameters into the logical functions of the BNs. This approach enables alteration of network behavior without needing detailed knowledge of the logical functions. An admissible use case for this approach is the adaptation of a “generic” BN to different “contexts,” such as for network personalization (Béal *et al.* 2019, 2021, Montagud *et al.* 2022).

In some studies, the notion of parameterized BNs is used for partially defined BNs, where for some variables, the logical functions are not defined due to the lack of evidence (Zou 2013, Brim *et al.* 2021). However, our focus is on the BNs that are completely defined, and the purpose of parameterization is to include stochastic noise in logical functions.

We developed pastboon, an R package to simulate parameterized stochastic Boolean networks. In the first version of the package, we implemented three methods to incorporate stochastic noise into the BNs. Specific code sections (mostly, the C codes) were taken from the BoolNet package (Müssel *et al.* 2010) and adapted to implement the methods included in our package.

## 2 Implementation

### 2.1 Basic definitions

A BN is characterized by n binary variables (nodes) and the Boolean logic update rules governing their dynamics. At a certain time-step t, the state of the ith node, $S_{t}^{\left(i\right)}$ can be either on (1) or off (0), where i∈{1,2,…,n}. The system’s state as a whole at the time-step t can be represented as:

(1) $$S_{t}={\left[S_{t}^{\left(1\right)}S_{t}^{\left(2\right)}\cdots S_{t}^{\left(n\right)}\right]}^{T}.$$

Thus, in general, the system can be in one of $2^{n}$ possible states. The temporal evolution of each variable i∈{1,2,…n} is governed by its predefined update rule,

(2) $$S_{t}^{\left(i\right)}=f_{i}(S_{t-1})=f_{i}\left({\left[S_{t-1}^{\left(1\right)}S_{t-1}^{\left(2\right)}\cdots S_{t-1}^{\left(n\right)}\right]}^{T}\right)$$

where $f_{i}$ defines the dynamics of the ith Boolean variable over time, as a function of the previous state of the system. Consequently, based on (2), the state vector of the BN at the time-step t can be written as $S_{t}=f\left(S_{t-1}\right)$. A node that is not influenced by any other node is called an “input node”. The input nodes can be further classified into two types: “identity nodes,” which are nodes that do not have update rules, or equivalently, have the identity function as their update rule, and “fixed nodes,” which are nodes whose update function is a fixed value. For more details, refer to Section S1 of the Supplementary Notes.

A BN can be either updated “synchronously,” i.e. at each time-step, all nodes are updated simultaneously, or “asynchronously,” i.e. at each time-step, a subset of nodes is updated, which may include one or more nodes. Moreover, if the next state of a BN is calculated deterministically based on its current state, it is called “deterministic”; otherwise, it is called “stochastic.”

For a BN with n nodes, one can consider a corresponding Markov chain as:

(3) $$\pi_{t}=M\pi_{t-1}$$

such that $\pi_{t}$ (respectively, $\pi_{t-1}$) is a vector of size $2^{n}$ where its ith element indicates the probability of presence in the state $S_{t}^{\left(i\right)}$ at the time-step t (respectively, t−1) (Stoll *et al.* 2012). The matrix M is the transition matrix with dimensions $2^{n}\times 2^{n}$, where each element $M_{ij}$ represents the probability of transitioning from the jth state to the ith state.

### 2.2 Parameterized stochastic BN models

In pastboon, three parameterization methods have been implemented. The “Boolean network with perturbation” (BNp) method (Trairatphisan *et al.* 2013) is one of the most basic techniques to include stochasticity into BNs. Based on this method, at each time-step t, the ith node can either be flipped with probability $p_{i}$ or updated based on its original update rule. Formally, the update rule of the ith variable is redefined as:

(4) $$S_{t}^{\left(i\right)}=\{\begin{array}{cc} \neg S_{t-1}^{\left(i\right)} & :\;\text{with}\;\text{probability}\;p_{i}\\ f_{i}(S_{t-1}) & :\;\text{with}\;\text{probability}\;1-p_{i} \end{array}.$$

When $p_{i}\in \left(0,1\right)$, the Markov chain corresponding to the BN is guaranteed to be ergodic, i.e. every state is reachable from any other state.

The second implemented method is “stochastic discrete dynamical systems” (SDDS), which uses another strategy to incorporate stochasticity into BNs (Murrugarra *et al.* 2012). In SDDS, two “parameters,” namely, $p_{i}^{↑}$ (activation propensity) and $p_{i}^{↓}$ (degradation propensity) are considered in updating the state of each node. These two parameters, $p_{i}^{↑}$ and $p_{i}^{↓}$, are, respectively, equivalent to $\rho_{\mathrm{off}\to \mathrm{on}}^{\left(i\right)}$ and $\rho_{\mathrm{on}\to \mathrm{off}}^{\left(i\right)}$, defined as:

(5) $$\begin{array}{c} \rho_{\text{off}\to \text{on}}^{\left(i\right)}:=P\left(S_{t}^{\left(i\right)}=\text{on}|S_{t-1}^{\left(i\right)}=\text{off},f_{i}\left(S_{t-1}\right)=\text{on}\right),\\ \rho_{\text{on}\to \text{off}}^{\left(i\right)}:=P\left(S_{t}^{\left(i\right)}=\text{off}|S_{t-1}^{\left(i\right)}=\text{on},f_{i}\left(S_{t-1}\right)=\text{off}\right). \end{array}$$

We implemented a generalization of the SDDS model by assigning two additional parameters for preserving the value of each node i, that is, $\rho_{\mathrm{off}\to \mathrm{off}}^{\left(i\right)}$ and $\rho_{\mathrm{on}\to \mathrm{on}}^{\left(i\right)}$:

(6) $$\begin{array}{c} \rho_{\text{off}\to \text{off}}^{\left(i\right)}:=P\left(S_{t}^{\left(i\right)}=\text{off}|S_{t-1}^{\left(i\right)}=\text{off},f_{i}\left(S_{t-1}\right)=\text{off}\right),\\ \rho_{\text{on}\to \text{on}}^{\left(i\right)}:=P\left(S_{t}^{\left(i\right)}=\text{on}|S_{t-1}^{\left(i\right)}=\text{on},f_{i}\left(S_{t-1}\right)=\text{on}\right). \end{array}$$

The original SDDS model is a special case of our implemented model, where $\rho_{\mathrm{on}\to \mathrm{on}}^{\left(i\right)}=\rho_{\mathrm{off}\to \mathrm{off}}^{\left(i\right)}=1$. When all propensities are chosen from the values in the range (0,1), the ergodicity of the model is ensured. For more details, see Section S2 of the Supplementary Notes.

The third implemented method is “probabilistic edge weights” (PEW) (Deritei *et al.* 2022), in which each hyper-edge, rather than node, is assigned on and off probabilities to adjust its “strength.” In the original reference (Deritei *et al.* 2022), a hyper-edge is defined as a clause within Boolean functions, where multiple nodes collectively regulate the same target node. In the current implementation of the package, noise is applied to edges, i.e. input variables in a logical equation, rather than hyper-edges. Therefore, for each input variable of each function, we define two parameters, $p_{\mathrm{on}}$ and $p_{\mathrm{off}}$, which determine the strength of that particular edge. More precisely, one can define operator $P_{e}$ as:

(7) $$P_{e}S_{t-1}^{\left(i\right)}=\left\{ \begin{array}{cc} g_{\mathrm{on}}(p_{\mathrm{on}}^{\left(i\right)}) & :\;\text{if}\;S_{t-1}^{\left(i\right)}=\mathrm{on}\\ g_{\mathrm{off}}(p_{\mathrm{off}}^{\left(i\right)}) & :\;\text{otherwise} \end{array}\right.$$

such that each of the functions $g_{\mathrm{on}}$ and $g_{\mathrm{off}}$ yields the outcome of a simple draw from a Bernoulli distribution, similar to the outcome of tossing a coin with the bias $p_{\mathrm{on}}$ or $p_{\mathrm{off}}$:

(8) $$\begin{array}{c} g_{\mathrm{on}}(p_{\mathrm{on}})=\left\{ \begin{array}{cc} \text{on} & :\;\text{with}\;\text{probability}\;p_{\mathrm{on}}\\ \text{off} & :\;\text{with}\;\text{probability}\;1-p_{\mathrm{on}} \end{array}\right.,\\ g_{\mathrm{off}}(p_{\mathrm{off}})=\left\{ \begin{array}{cc} \mathrm{off} & :\;\text{with}\;\text{probability}\;p_{\mathrm{off}}\\ \text{on} & :\;\text{with}\;\text{probability}\;1-p_{\mathrm{off}} \end{array}\right.. \end{array}$$

By applying $P_{e}$ on (2), we get:

(9) $$P_{e}S_{t}^{\left(i\right)}=f_{i}\left({\left[P_{e}S_{t-1}^{\left(1\right)}P_{e}S_{t-1}^{\left(2\right)}\cdots P_{e}S_{t-1}^{\left(n\right)}\right]}^{T}\right).$$

When there are no fixed nodes, setting all strength parameters, $p_{\mathrm{on}}^{\left(i\right)}$ and $p_{\mathrm{off}}^{\left(i\right)}$, to values in the range (0,1) ensures ergodicity of the system. For more details, see Section S3 of the Supplementary Notes.

Note that SDDS and PEW models have been proposed as general discrete dynamical systems (i.e. having variables with more than two levels). However, our current pastboon implementation focuses on binary variables only.

### 2.3 Package functionalities

The pastboon package offers functions to study BNs using the three discussed parameterization methods: BNp, SDDS, and PEW. Table 1 summarizes these functions. Core parts of functions calc_node_activities, count_pairwise_trans, and get_reached_states have been implemented in C for faster simulations. The functions that receive a network as input require a network of class BooleanNetwork from the BoolNet package (Müssel *et al.* 2010).

**Table 1.** List of functions in the pastboon package.

Function	Description
calc_node_activities	Calculates the average activity of each node
calc_convergence_time	Calculates convergence time for node activities
count_pairwise_trans	Counts the transitions between states
extract_edges	Extracts the edges from a given network
get_reached_states	Obtains the reached states after simulation

**Figure 1.**
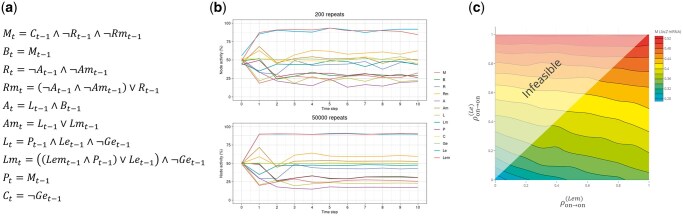
(a) Update rules governing node states in the *lac* operon BN model. Since the nodes *Ge*, *Le*, and *Lem* are input nodes, no update rules are assigned to them, which is equivalent to having identity function as their update rule. **(b)** Time-series plot of node activities using SDDS with 200 and 50 000 repeats. All parameters were set to 0.9, all initial probabilities were set 0.5, and synchronous updating was used. **(c)** Effect of $\rho_{\text{on}\to \text{on}}^{\left(Le\right)}$ and $\rho_{\text{on}\to \text{on}}^{\left(\mathrm{Lem}\right)}$ over *lacZ* mRNA activity (M) at the time-step t=10 using 50 000 repeats, when other parameters were chosen according to Table 2, the input nodes were fixed as $Ge_{t}=\text{off}$, $Le_{t}=\mathrm{on}$, $Lem_{t}=\mathrm{on}$, and the initial probabilities were chosen as 0 for the input nodes and 0.5 for the other nodes.

In Section S4 of the Supplementary Notes, we provided examples of all functions and parameterization methods on the Boolean models of *lac* operon (Veliz-Cuba and Stigler 2011) and myeloid differentiation (Krumsiek *et al.* 2011) gene regulatory networks. Here, we describe the package functions and demonstrate an example using the SDDS method with the *lac* operon BN to show a use case of the package. The *lac* operon BN model consists of 13 nodes, among which our focus is on the nodes *Ge* (extracellular glucose concentration), *Le* (extracellular lactose high concentration), *Lem* (extracellular lactose medium concentration), and *M* (*lacZ* mRNA). The evolution rules of the *lac* operon BN are presented in Fig. 1a.

The function calc_node_activities calculates each node’s activity rate (i.e. average state) at each time-step by simulating the evolution of network state multiple times. Users can specify the parameterization method (BNp, SDDS, or PEW), parameter values, number of time-steps and repetitions, initial activity probabilities for each node, whether to return the node activities across all time-steps (as a time-series) or only the final time-step, the updating scheme (synchronous or asynchronous), and the probability of updating each node in asynchronous cases. The averaging of node states converts a discrete binary variable, X∈{0,1}, into a continuous variable $\hat{X}\in \left[0,1\right]$ (Albert and Thakar 2014). In this context, the continuous variable $\hat{X}$ represents the average activity level of the node X at a specific time-step over multiple repetitions.

For large networks, calculating and storing a probability distribution over the entire state space is infeasible due to the exponential growth of the number of states with the number of variables. Meanwhile, node activity rates, essentially the marginal probabilities of the variables, provide an informative representation of a probability distribution across all states and can be calculated and stored for large networks.

Figure 1b shows the node activity time-series for the *lac* operon BN using the SDDS method with synchronous update. The initial node activity probabilities were set to 0.5, and all parameters were set to 0.9 to ensure ergodicity, making the steady-state activity profile independent from initial node activities. Figure 1b shows that increasing the number of repeats leads to smoother activity curves. The input variables (*Ge*, *Le*, and *Lem*) maintain an average activity level of ∼50% across all time-steps, which can be attributed to the initial on/off probability of 0.5 and the identity function update rule. Additionally, node activities rapidly reach visible stability by time-step 5.

Calculation of average node activities provides insight into network behavior under varying parameter values. For instance, for the *lac* operon BN, Fig. 1c illustrates the steady-state activity of the node M (*lacZ* mRNA) for different values of $\rho_{\text{on}\to \text{on}}^{\left(Le\right)}$ and $\rho_{\text{on}\to \text{on}}^{\left(\mathrm{Lem}\right)}$ in the form of a contour plot, based on the SDDS method, while the parameters were chosen according to Table 2, the input nodes were fixed as $Ge_{t}=\text{off}$, $Le_{t}=\text{on}$, $Lem_{t}=\text{on}$, and the initial probabilities were chosen as 0 for the input nodes and 0.5 for the other nodes. Figure 1c shows greater influence of the node *Le* on *M* compared to that of *Lem* as the activity of *M* seems to vary more with *Le*.

**Table 2.** Values of the parameters for the input nodes based on the SDDS method to generate the contour plot of Fig. 1.

Node name	$\rho_{\text{off}\to \text{off}}$	$\rho_{\text{off}\to \text{on}}$	$\rho_{\text{on}\to \text{off}}$	$\rho_{\text{on}\to \text{on}}$
*Ge*	1	∗	∗	∗
*Le*	∗	1	∗	†
*Lem*	∗	1	∗	†
Other nodes	0.9	0.9	0.9	0.9

∗ Any value in [0,1].

^†^For each value in {0, 0.1, … ,1.0}.

The calc_convergence_time function estimates the time-step at which the steady-state distribution is reached, utilizing the activity time-series calculated by calc_node_activities. This function requires the change threshold and window size as input parameters. The convergence criterion entails the difference in mean activity values between two same-sized time windows ending at t and t−1 being less than a user-defined threshold for all variables.

The count_pairwise_trans function, for each state pair S, T in a given set of states, determines the frequency of transitions from S to T and vice versa for a specified maximum number of time-steps and repeats. The function’s arguments enable customization of parameterization method, parameter values, set of states, number of time-steps and repeats, updating scheme, and each node’s update probability when utilizing asynchronous updates. This function serves as a tool to estimate the reachability of each state from the other states.

The extract_edges function retrieves the directed edges from a given BN. This function serves as a particularly useful tool when employing the PEW parameterization method, which necessitates the assignment of two parameters $p_{\mathrm{on}}$ and $p_{\mathrm{off}}$, to each edge.

Finally, the get_reached_states function determines the reached states after simulating a network for a specified number of time-steps and repeats. The function’s arguments enable customization of the parameterization method, parameter values, number of time-steps and repeats, set of initial states, updating scheme, and each node’s update probability when utilizing asynchronous updates.

### 2.4 Availability

The package pastboon is freely available from R CRAN repository at https://cran.r-project.org/package=pastboon and can be installed using the command install.packages(“pastboon”). The pastboon’s source code is also available on GitHub at https://github.com/taherimo/pastboon.

## 3 Conclusion

The pastboon package provides a suite of functions that facilitate the analysis of parameterized stochastic BNs across diverse parameter values. This tool enables users to explore and understand the influence of various parameter configurations on the behavior of BNs, such as the phenotypic effects of different perturbations on BN models describing cellular processes. A key application of pastboon entails tailoring a generic BN that describes molecular mechanisms to specific contexts (cell-types, conditions, etc.) where each context is represented by a noise pattern. It allows researchers to analyze unique scenarios in biology, such as gene regulatory networks in different cell-types or conditions (Béal *et al.* 2019, 2021, Montagud *et al.* 2022).

## Supplementary Material

vbaf017_Supplementary_Data

## Data Availability

The two example Boolean networks used in this paper, the *lac* operon and myeloid differentiation gene regulatory networks, are included in the R package.
