# Supplementary material for: pastboon: an R package to simulate parameterized stochastic Boolean networks
Source: Bioinform Adv. 2025 Feb 6;5(1):vbaf017. doi: 10.1093/bioadv/vbaf017 (PMC12007881; doi:10.1093/bioadv/vbaf017)
Supplement: vbaf017_Supplementary_Data [file vbaf017_supplementary_data.pdf]

pastboon: an R package to simulate parameterized stochastic Boolean networks

Supplementary Notes

Mohammad Taheri-Ledari, Sayed-Amir Marashi, and Kaveh Kavousi

S1 Additional definitions

As described in the main text, the *input nodes* of a Boolean network (BN) are defined as nodes that are not influenced by the state of any other nodes. The input nodes can be further classified into two types: *identity nodes*, which are nodes that do not have update rules, or equivalently, have the identity function as their update rule (i.e., $s_t^{(i)} = s_{t-1}^{(i)}$ for the node i), and *fixed nodes*, which are nodes whose update function is a fixed value (i.e., either $s_t^{(i)} = \mathsf{on}$ or $s_t^{(i)} = \mathsf{off}$ for node i).

Here, we introduce two additional terms to provide a more comprehensive classification of nodes in BNs. *Output nodes* are the nodes that do not exert any influence or regulatory effect on other nodes within the network. Furthermore, nodes that are neither input nor output nodes are called *internal nodes*. These nodes participate in regulatory interactions of the network by affecting and being affected by the other nodes.

To distinguish input, output, and internal nodes, one can inspect the directed graph representation of the BN. In such a graph, an input node has no incoming edge from the other nodes, while an output node has no outgoing edges to the other nodes. An identity node appears as a node with self-loop as its only incoming edge.

Considering the above definitions, every type of node (including input nodes) has an update function and can be updated at each time-step. An identity node is updated to its previous value and a fixed node is updated to a fixed value on/off.

S2 Existing tools to simulate and analysis of stochastic BNs

Unlike deterministic BNs, which evolve predictably based on fixed rules, stochastic BNs introduce stochasticity (randomness) into their state transitions. This randomness can arise from intrinsic noise in biological processes, such as molecular fluctuations, or from external factors, such as environmental variability. The state of each node in the network is updated based on logical rules influenced by the states of neighboring nodes, with probabilistic transitions that capture uncertainty.

Stochastic BNs are widely used in modeling dynamic behaviors in biological systems, such as gene regulatory networks, cell signaling pathways, and neural circuits. For example, they can simulate how a gene's expression level may fluctuate due to stochastic biochemical reactions. These models are particularly valuable for studying systems where noise plays a critical functional role, such as bistable switches, oscillatory systems, and decision-making processes in cells. Beyond biology, stochastic BNs are applied in fields like social network analysis, where they can model opinion dynamics with

Table S1: Tools for simulating and analyzing stochastic BNs.

Tool	Description	Platform	Year	Citation
MaBoSS	Software for simulating BN by applying Gillespie algorithm on BNs which two transition rates are assigned each node.	Linux-based tool	2017	[1, 2]
BoolFilter	Implements partially-observed Boolean dynamical systems with stochastic noise.	R package	2017	[3]
Cell Collective	Online platform for modeling and simulating biological networks, including BNs with stochastic asynchronous update.	Web-based application	2012	[4]
BoolNet	R package for constructing, simulating, and analyzing BNs. It supports simulation of probabilistic BNs (proposed by Shmulevich et al.) and stochastic asynchronous update.	R package	2010	[5]
BooleanNet	Python package for BN modeling and simulation which supports stochastic asynchronous update.	Python package	2003	[6]

probabilistic interactions, and in engineering, for fault-tolerant systems and network reliability studies. Their ability to incorporate randomness makes stochastic BNs a powerful tool for exploring the complex and often unpredictable dynamics of real-world systems. Table S1 lists the existing tools to simulate stochastic BNs. Before developing the `pastboon` package, there was no tool that integrated parameterization methods to induce noise into BNs. Also, no dedicated R package has existed to use such methods.

S3 Supplementary descriptions

Although all three parameterization methods implemented in `pastboon` incorporate stochastic noise into BNs, they are different in nature. In particular, the BNp method is used to control the degree of chaosity, while the SDDS and PEW methods are used to control the tendency of the nodes and edges to be active. In Table S3, the purpose, strengths and weaknesses of the three implemented methods are summarized. In addition, Table S2 lists the names and descriptions of the functions implemented in the package along with a column showing whether each function includes network simulation or not.

Table S2: List of functions in the `pastboon` package.

Function	Description	Includes simulation
`calc_node_activities`	Calculates the average activity of each node	Yes
`calc_convergence_time`	Calculates convergence time for node activities	No
`count_pairwise_trans`	Counts the transitions between states	Yes
`extract_edges`	Extracts the edges from a given network	No
`get_reached_states`	Obtains the reached states after simulation	Yes

In all the implemented methods, there is a parameter setting based on which the network acts according to its original update rules. More precisely, in the BNp method, if all parameters are set to 0, and in the SDDS and PEW methods, if all parameters are set to 1, the network acts based on its original logical rules. Given a BN and its current parameter values p, one can interpret a perturbation as a change in parameter values Δp such that the sum of $p + \Delta p$ changes the behavior of the network. A classical problem in control theory [7] is finding an optimal perturbation vector Δp to push the system from an undesired behavior to a desired behavior in terms of some objective functions.

S3.1 Automatic determination of the number of repeats

Every function that involves simulations has a "repeats" argument, which determines the number of network simulations. One way to automatically determine the number of repeats is to start with an arbitrary number of repeats with the `calc_node_activities` function and check whether the smoothness of the resulting time-series is sufficient or not. If the smoothness of the time-series is not good enough, the number of repeats is incremented. This procedure is continued until the desired level of smoothness is reached. To measure smoothness of a time-series one can calculate the standard deviation of the differences, i.e., standard deviation of $x_2 - x_1, x_3 - x_2, \cdots, x_T - x_{T-1}$ where x_t is activity of the node x at the time-step t and T is the number of time-steps.

As an example, the following code determines the number of repeats based on the aforementioned approach over a random BN:

```
library(pastboon)
library(BoolNet)

net <- generateRandomNKNetwork(10, 6, "scale_free")

reps <- 1
smoothness <- 1

while (smoothness > 0.01) {
  act <- calc_node_activities(net, method = "BNp",
      params = rep(0.1,length(net$genes)), steps = 100,
      repeats = reps)

  reps <- reps + 1
  smoothness <- mean(apply(diff(act), 2, sd))
}

print(reps)
```

Table S3: Comparison of the implemented methods.

Method	Purpose	Strengths	Weaknesses
BNp	Controlling the degree of chaos in node state	Simplicity and ability to guarantee ergodicity	Inability to control the tendency of variables to be active
SDDS	Controlling the tendency of the nodes to be active	Ability to control the tendency of variables to be active and to guarantee ergodicity	Intricacy of modulating parameter values
PEW	Controlling the tendency of the edges to be active	Ability to control the tendency of interactions (edges) to be active	Inability to guarantee ergodicity in the case of having fixed nodes

S3.2 Time efficiency and scalability

A simulation involves updating the state of a BN over a specified number of time-steps. In the `pastboon` package, simulating BNs is the bottleneck for calculation speed. To address this, we implemented simulations in `C`, with the initial code adapted from the `BoolNet` package [5]. Theoretically, for a standard BN of size n with any topology, assuming that updating a single node takes a fixed time c, the time required depends on the update scheme. In the synchronous update scheme, all n nodes are updated at each time-step, which requires $c \times n$ time per step. Thus, a simulation with t time-steps and r repeats takes $t \times r \times n \times c$ time. In contrast, the asynchronous update scheme updates only one node per time-step, resulting in a total simulation time of $t \times r \times c$. Therefore, in the asynchronous

case, the time complexity is independent of the network size. However, the size of the network can influence the update time c, as larger networks can include nodes with a higher number of inputs.

To measure the time of simulating networks of different sizes, we performed a computational experiment for the `calc_node_activities` function over random BNs using the three parameterization methods and the synchronous and asynchronous update schemes. To do so, we generated 20 random scale-free networks using `BoolNet`'s `generateRandomNKNetwork` function which takes the arguments n and k as network size and maximum node degree. We chose n from the values 5 to 100 with the interval 5. In each case, we randomly selected a value between $0.4 \times n$ to $0.6 \times n$ as the k argument. Then, we measured the execution time for running the function `calc_node_activities` with 50,000 repeats and 300 time-steps in the two synchronous and asynchronous cases. The experiment was performed on an Ubuntu machine with a Xeon E5-2699 2.20 GHz processor. Figure S1 shows the execution times for different combinations of the parameterization methods and update schemes. According to Fig. S1, the time grows linearly with the size of the network in the synchronous case. Also, we don't observe much change in execution time based on network size when using asynchronous update. These results are in agreement with our theoretical analysis of the time complexity of the simulations. In addition, BNp seems to be significantly faster than SDDS and PEW. The PEW method shows slower execution speed, which seems to be a result of checking each input edge when updating a node.

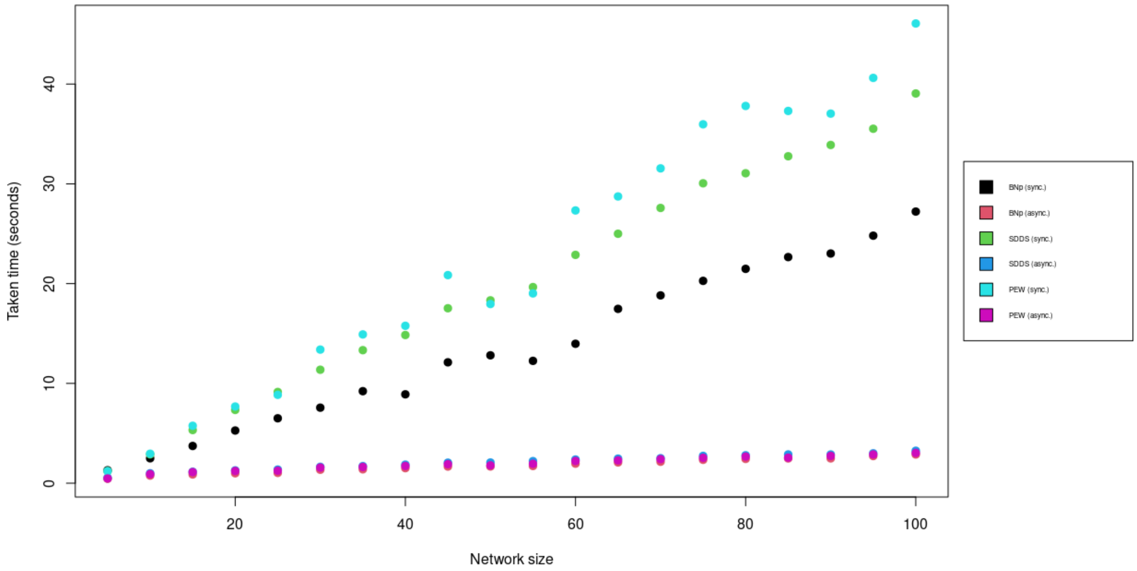

Figure S1: The time of running the `calc_node_activities` function with 50,000 repeats and 300 time-steps using asynchronous update over random netwrorks.

A simulation results in a sequence of states, i.e., state trajectory. Storing r simulations for t time-steps for a network of size n in memory takes $t \times r \times n$ bits. Although it is linear with respect to t, r, and n, but since it can lead to a huge value for large values of t, r, and, n, in the current version of the package, the simulation trajectories are not stored in memory. Thus, if the user calls two functions that involve simulations one after the other, the simulations are repeated.

As mentioned in the main text, for a BN of size n, storing a probability distribution over the entire state space requires storing 2^n real numbers in memory, which becomes infeasible for large networks. However, node activity rates provide estimations of the marginal probabilities of the variables. By

storing the n activity rates, which is feasible even for large networks, we can gain good insight from the probability distribution across all network states. Starting a network from a given marginal probability profile (initial probabilities) allows the calculation of joint probabilities, which, for an ergodic network, will converge to a unique distribution.

S3.3 Supplementary descriptions about the SDDS method

Murrugarra and his colleagues proposed the SDDS method [8] to model stochasticity in discrete dynamical models of gene regulatory networks. To this end, they assigned two parameters to each node: $p_i^{\uparrow}$ (activation propensity), defined as the probability that node i will be updated based on its logical rule if the update leads to an increase of the variable, and $p_i^{\downarrow}$ (degradation propensity), defined as the probability that node i will be updated based on its logical rule in case the variable is decreased. In the present work, we assume that all variables are Boolean. Consequently, we denote the parameters $p_i^{\uparrow}$ and $p_i^{\downarrow}$ with $\rho^{(i)}_{\mathsf{off}\to\mathsf{on}}$ and $\rho^{(i)}_{\mathsf{on}\to\mathsf{off}}$, respectively.

It should be noted that $\rho^{(i)}_{\mathsf{off}\to\mathsf{on}}$ and $\rho^{(i)}_{\mathsf{on}\to\mathsf{off}}$ determine the characteristics of the SDDS model in a non-trivial manner. Figure S2 shows the effect of different values of $\rho^{(i)}_{\mathsf{off}\to\mathsf{on}}$ and $\rho^{(i)}_{\mathsf{on}\to\mathsf{off}}$ on node i. There are five important cases:

- Point **A**: when $\rho^{(i)}_{\mathsf{off}\to\mathsf{on}} = \rho^{(i)}_{\mathsf{on}\to\mathsf{off}} = 1$, the original deterministic transition is applied.
- Point **B**: when $\rho^{(i)}_{\mathsf{off}\to\mathsf{on}} = \rho^{(i)}_{\mathsf{on}\to\mathsf{off}} = 0$, the negation of the original deterministic transition is applied.
- Point **C**: when $\rho^{(i)}_{\mathsf{off}\to\mathsf{on}} = 0$ and $\rho^{(i)}_{\mathsf{on}\to\mathsf{off}} = 1$, the update rule of node i is fixed to off.
- Point **D**: when $\rho^{(i)}_{\mathsf{off}\to\mathsf{on}} = 1$ and $\rho^{(i)}_{\mathsf{on}\to\mathsf{off}} = 0$, the update rule of node i is fixed to on.
- Point **E**: when $\rho^{(i)}_{\mathsf{off}\to\mathsf{on}} = \rho^{(i)}_{\mathsf{on}\to\mathsf{off}} = 0.5$, the update rule of node i is determined randomly.

As a general guide for choosing parameter values, the closer we choose values to the dotted red line in Fig. S2, the less dependent the new state of the variable will be on its current state. Therefore, a general good practice to choose parameter values is to choose the values over the top and right boundaries where, although the tendency of the variable to be active or inactive can be controlled, at the same time the Markovian property of the variable (i.e., dependency of the next state to the current state) is preserved.

In the SDDS method, when all variables are binary, $\rho^{(i)}_{\mathsf{off}\to\mathsf{on}}$ (respectively, $\rho^{(i)}_{\mathsf{on}\to\mathsf{off}}$) can be interpreted as the probability that the output of the logical function for node i is negated if it results in an increase (respectively, decrease) of the variable. In other words, in the case of binary variables, if the current state of node i is off (respectively, on) and by applying the update function its next state will be on (respectively, off), then tossing a coin with bias $\rho^{(i)}_{\mathsf{off}\to\mathsf{on}}$ (respectively, $\rho^{(i)}_{\mathsf{on}\to\mathsf{off}}$) determines whether the next state should be the output of the update function, or its negation.

With the above interpretation, we generalize the SDDS method by introducing the concepts of "inaction" and "operation" propensities, as follows. For node i, the inaction propensity ($\rho^{(i)}_{\mathsf{off}\to\mathsf{off}}$) is the probability that the output of the logical function of node i is preserved (not negated) if the function causes node i to remain inactive, while the operation propensity ($\rho^{(i)}_{\mathsf{on}\to\mathsf{on}}$) is the probability that the output of the logical function of node i is preserved if the function causes node i to remain active. The addition of these two parameters for each node i aims to provide more control over the

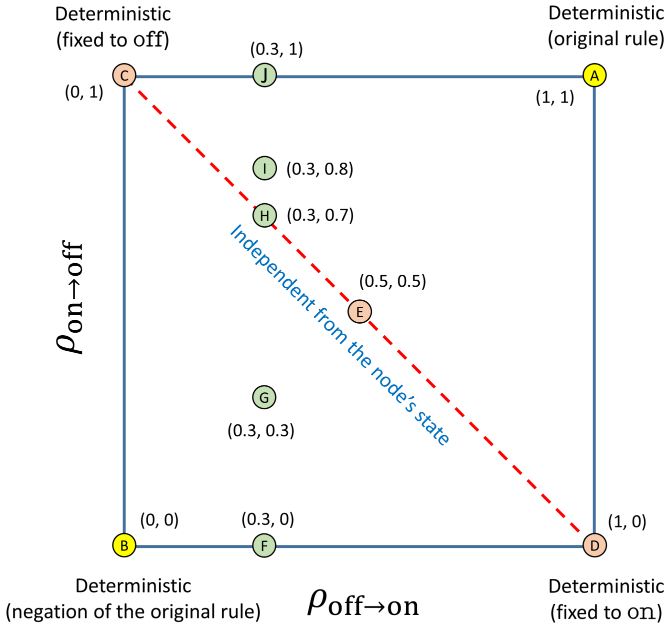

Figure S2: The parameter space of $\rho^{(i)}_{\mathsf{off}\to\mathsf{on}}$ and $\rho^{(i)}_{\mathsf{on}\to\mathsf{off}}$ for a node based on the SDDS method. The points A and B lead to deterministic updating based on the original update rule and the negation of the original update rule, respectively. The points on the dashed line indicate that under the corresponding parameter settings, the state of the node is determined randomly (based on a coin toss, independent of the node's state). Two points, C and D, represent extreme cases corresponding to fixing the node to off and on, respectively. Point E, the midpoint of the dashed line, represents the conditions where the node's state is determined randomly, similar to tossing an unbiased coin. The parameter values at points F, G, H, and I mean that if the current state of the node is off and applying the original update rule would change it to on in the next step, then updating is applied with the probability of 0.3. Conversely, if the current state of the node is on and applying the original update rule would change it to off in the next time-step, for the parameter values corresponding to the points F, G, H, I, and J, the updating is applied with probabilities 0, 0.3, 0.7, 0.8, and 1, respectively.

noise effect in the cases off $\to$ off and on $\to$ on, which, along with the existing parameters $\rho^{(i)}_{\mathsf{off}\to\mathsf{on}}$ and $\rho^{(i)}_{\mathsf{on}\to\mathsf{off}}$, allow us to manage all possible transitions.

Also, as mentioned in Section S1, one can consider an update function for every type of node including identity nodes and fixed nodes. An identity node is updated to its previous value and a fixed node is updated to a fixed value on/off. Therefore, in the case of the SDDS method, if a node i is an identity node (i.e., its update function is $s^{(i)}_t = s^{(i)}_{t-1}$), only the parameters $\rho^{(i)}_{\mathsf{off}\to\mathsf{off}}$ (if the current state is off) or $\rho^{(i)}_{\mathsf{on}\to\mathsf{on}}$ (if the current state is on) are effective. Also, if a node i is a fixed node, we may have two possible update functions: $s^{(i)}_t = \mathsf{on}$ and $s^{(i)}_t = \mathsf{off}$. In the first case, only the parameters $\rho^{(i)}_{\mathsf{off}\to\mathsf{on}}$ and $\rho^{(i)}_{\mathsf{on}\to\mathsf{on}}$, and, in the second case, only the parameters $\rho^{(i)}_{\mathsf{off}\to\mathsf{off}}$ and $\rho^{(i)}_{\mathsf{on}\to\mathsf{off}}$ are effective.

S3.4 Supplementary descriptions about the PEW method

Deritei et al. proposed the PEW method [9] in which noise is applied to edges instead of nodes. While in a BN, each input variable in an update function can be considered an edge, any clause of a Boolean rule can be regarded as a hyper-edge to which noise is applied. In the current implementation of `pastboon`, noise is applied to each edge individually rather than to grouped edges as hyper-edges. In the PEW method, the parameters p_{on} and p_{off} are assigned to each edge. When calculating the state based on the update function, if the value of an edge is on, it remains on with the probability p_{on};

otherwise if the value of the edge is off, it remains off with the probability p_{off}. We can consider p_{on} and p_{off} as the weight (strength) of each edge. Figure S3 shows the effect of different values of p_{on} and p_{off} on an edge. There are five important cases:

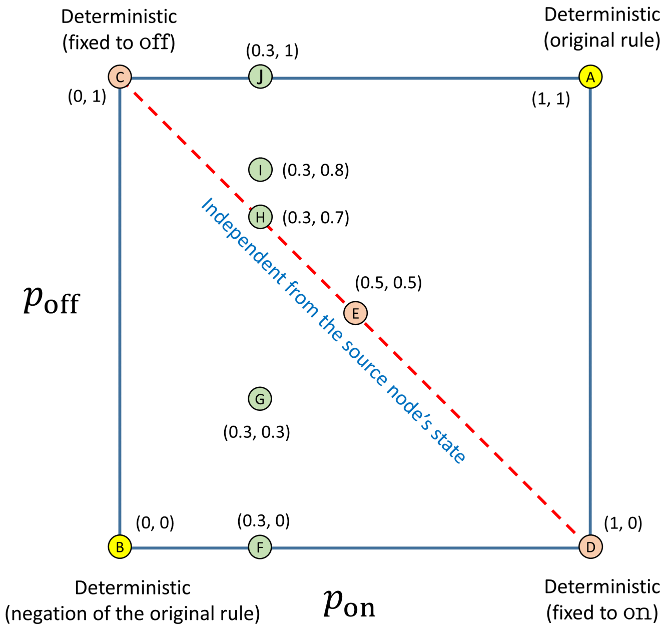

Figure S3: The parameter space of p_{on} and p_{off} for an edge based on the PEW method. The points A and B lead to the original deterministic functioning of the edge or its negation, respectively. The parameter values over the dashed line indicate that the value of the edge is determined randomly, based on a coin toss (independent from the source node's state) with two points C and D as extreme cases corresponding to fixing the edge to off and on, respectively. The center of the dashed line which is the point E, results in determining the edge's value by tossing a fair coin. In fact, the dashed line corresponds to minimum edge strength, which is equivalent to the elimination of the edge. The parameter values corresponding to the points F, G, H, and I, mean that if the value of the edge is on, then the edge remains on with the probability of 0.3. Meanwhile, if the value of the edge is off, for the parameter values corresponding to the points F, G, H, I, and J, the edge remains off with the probabilities 0, 0.3, 0.7, 0.8, and 1, respectively.

- Point **A**: when $p_{\text{on}} = p_{\text{off}} = 1$ the edge acts like the original deterministic case (maximum edge weight).
- Point **B**: when $p_{\text{on}} = 0$, $p_{\text{off}} = 1$ the edge is fixed to off regardless of the state the source node (minimum edge weight, i.e., the edge is eliminated).
- Point **C**: when $p_{\text{on}} = 1$, $p_{\text{off}} = 0$ the edge is fixed to on regardless of the state of the source node (minimum edge weight, i.e., the edge is eliminated).
- Point **D**: when $p_{\text{on}} = p_{\text{off}} = 0.5$ the value of the edge is determined by tossing a fair coin regardless of the state of the source node (minimum edge weight, i.e., the edge is eliminated).
- Point **E**: when $p_{\text{on}} = p_{\text{off}} = 0$ the edge acts based on the negation of the original deterministic case (maximum edge weight but negated).

Similarly to the SDDS method, the closer we choose values to the dotted red line in Fig. S3, the less dependent the new value of the edge will be on the state of its source variable. Thus, a general

good practice to choose parameter values is to choose the values over the top and right boundaries where, although the tendency of the edge to be active or inactive can be controlled, at the same time the dependency of the new value of the edge to the state of its source node is preserved.

As mentioned in Section S1, every type of node, including identity nodes and fixed nodes, can be updated. An identity node is updated to its previous value and a fixed node is updated to a fixed value on/off. Therefore, in the case of the PEW method, if a node i is an identity node (i.e., its update function is $s_t^{(i)} = s_{t-1}^{(i)}$), it means that there is a self-loop edge whose source and destination nodes are the same. In this case, the parameters p_{on} and p_{off} of the edge affect the state of the node. If a node i is a fixed node, it means that there are no incoming edges to the node, so we cannot influence the state of the node through the parameters.

S4 Examples from package functionalities

In this section we provide examples of the functionalities of `pastboon` over two published BN models: the *lac* operon gene regulatory network [10], and the myeloid differentiation gene regulatory network [11]. We have included `R` source codes to reproduce all results.

S4.1 The example of *lac* operon BN

In the main text, as an example, we demonstrated the use of `pastboon` to showcase how the SDDS method can be applied in studying the effect of lactose concentration on *lacZ* mRNA expression. This was done using a BN model of *Escherichia coli*'s *lac* operon gene regulatory network [10]. The network comprises ten internal nodes: M, B, R, Rm, A, Am, L, Lm, P, and C, along with three input nodes: Ge, Le and Lem.

Note that Rm, Am, Lm and Lem serve as "modifier variables". These modifiers enable us to incorporate multiple concentration levels and overcome the common binary assumption of off or on, or equivalently 0 or 1, for the corresponding component. Therefore, these modifier variables facilitate a more nuanced representation of gene expression in the model, beyond the standard binary assumption of only off or on. For example, in the case of the (R, Rm) pair, we can capture three distinct concentration levels of repressor protein: (0, 0) for "low", (0, 1) for "medium", and (1, 1) for "high" concentration levels (with $Rm \geq R$). Am, Lm and Lem are also defined in a similar way. By including such modifier variables, the model can better reflect the range of gene expression levels observed in biological systems.

The descriptions of the variables for the *lac* operon BN are shown in Table S4. The *lac* operon network consists of three structural genes: *lacZ* (encoding β-galactosidase, B), *lacY* (encoding lactose permease, P, for importing extracellular lactose, Le and Lem), and *lacA* (not included in the model). The operon is regulated by the *lacI* gene encoding the repressor of the operon (R and Rm). Upon increasing the concentration of intracellular lactose (L and Lm), it is partly converted to the inducer molecule, allolactose (A and Am). Allolactose acts as a signal to inactivate the repressor protein, allowing transcription of *lacZ* mRNA (M) and other *lac* operon genes. In the absence of glucose in growth medium (Ge), the presence or absence of extracellular lactose affects gene expression by modulating the activity of the repressor protein. As mentioned in the main text, the nodes Ge, Le, and Lem are input nodes. The update rules governing the states of the ten internal nodes are specified as follows:

Table S4: The description of *lac* operon network variables.

Variable name	Concentration of:
M	mRNA of *lacZ*
B	β-galactosidase (LacZ protein)
R	Repressor (LacI protein)
Rm	Repressor (medium level)
A	Allolactose
Am	Allolactose (medium level)
L	Lactose
Lm	Lactose (medium level)
P	Lactose Permease (LacY protein)
C	CAP (catabolite activator protein)
Ge	Glucose (extracellular)
Le	Lactose (extracellular)
Lem	Lactose (extracellular, medium level)

$$
\begin{aligned}
M_t &= C_{t-1} \wedge \neg R_{t-1} \wedge \neg Rm_{t-1} \\
B_t &= M_{t-1} \\
R_t &= \neg A_{t-1} \wedge \neg Am_{t-1} \\
Rm_t &= (\neg A_{t-1} \wedge \neg Am_{t-1}) \vee R_{t-1} \\
A_t &= L_{t-1} \wedge B_{t-1} \\
Am_t &= L_{t-1} \vee Lm_{t-1} \\
L_t &= P_{t-1} \wedge Le_{t-1} \wedge \neg Ge_{t-1} \\
Lm_t &= ((Lem_{t-1} \wedge P_{t-1}) \vee Le_{t-1}) \wedge \neg Ge_{t-1} \\
P_t &= M_{t-1} \\
C_t &= \neg Ge_{t-1}
\end{aligned}
\tag{S1}
$$

As previously mentioned, Ge, Le, and Lem are input nodes. Since no update rule is assigned to these nodes, in this specific case, we can treat them as identity nodes, which implies that their update rules follow the identity function:

$$Ge_t = Ge_{t-1}, \quad Le_t = Le_{t-1}, \quad Lem_t = Lem_{t-1} \tag{S2}$$

We set the random seed to the fixed value of 912 as follows so that the users can reproduce all results:

```
set.seed(912)
```

Also, we need packages `pastboon` and `plotly` to be loaded:

```
library(pastboon)
library(BoolNet)
library(plotly)
```

Next, we load the *lac* operon BN using the following command:

```
data(lac_operon_net)
```

Moreover, to plot activity of the nodes, we first generate a set of distinct colors (equal to the number of variables):

```
generate_colors <- function(n) {
    hues <- seq(0, 1, length.out = n + 1)[-1]
    s <- 0.8
    v <- 0.6
    colors <- hsv(h = hues, s = s, v = v)
    return(colors)
}

col_vec <- generate_colors(length(lac_operon_net$genes))
col_vec <- col_vec[sample(1:length(col_vec))]
```

Then, we define the following function for the purpose of visualizing activity time-series:

```
plot_node_activities <- function(node_activities, xlab, ylab) {
  old_par <- par(no.readonly = TRUE)
  layout(matrix(c(1, 2), nrow = 1), width = c(4, 1))
  par(mar = c(5, 4, 4, 0))
  matplot(1:nrow(node_activities), node_activities, type = "l",
      frame = TRUE, lwd = 2, lty = 1, xlab = xlab, ylab = ylab,
      col = col_vec)
  par(mar = c(5, 0, 4, 2))
  plot(c(0, 1), type = "n", axes = FALSE, xlab = "")
  legend("center", colnames(node_activities), col = col_vec,
         cex = 0.5, fill = col_vec)
  layout(matrix(1))
  par(old_par)
}
```

We first use the BNp method to obtain the activity time-series of the nodes for 20 time-steps using synchronous update and 50,000 repeats. To guarantee ergodicity of the network (to avoid from getting stuck in an attractor state), we set all parameter values to 0.1 (refer to the main text for description of the BNp method). The following code produces and plots the activity time-series of the nodes using the mentioned parameter setting:

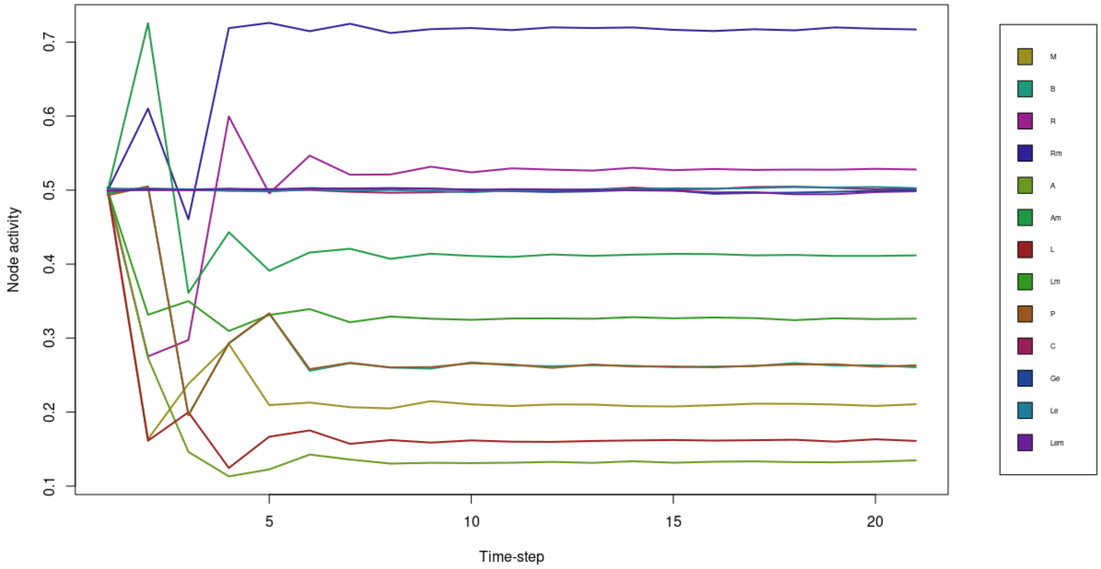

Figure S4: Time-series of the activity of *lac* operon network nodes for 20 time-steps and 50,000 repeats resulted from the `calc_node_activities` function with the BNp method where all parameters were set to 0.1 and synchronous update is used.

```
act <- calc_node_activities(lac_operon_net, method = "BNp",
    params = rep(0.1, length(lac_operon_net$genes)), steps = 20,
    repeats = 50000, asynchronous = F)

plot_node_activities(act, xlab = "Time-step", ylab = "Node activity")
```

Since the default updating scheme in `pastboon` is asynchronous update, we needed to set the "asynchronous" argument to FALSE. Figure S4 presents the resulting plot. Now, we can estimate steady-state time-step using the following command:

```
calc_convergence_time(act, 0.01, window_size = 3)
```

The above function returns 10 as the time-step at which the steady-state distribution of the network is reached. To emulate the condition of availability of glucose and lack of lactose, we set the inputs as $\mathit{Ge}_t = \text{on}$, $\mathit{Le}_t = \text{off}$, and $\mathit{Lem}_t = \text{off}$:

```
input_idx <- c(which(lac_operon_net$genes == "Ge"),
    which(lac_operon_net$genes == "Le"),
    which(lac_operon_net$genes == "Lem"))

lac_operon_net_glucose <- fixGenes(lac_operon_net, input_idx, c(1, 0, 0))
```

With the mentioned condition, we are going to study which states are reached most from different random starting states. Here, we use the package's `get_reached_states` function with 10 time-steps (steady-state) and 10,000 repeats. The rest of the arguments are set same as the setting of `calc_node_activities` function. Since all parameters related to the BNp method are set to 0.1, the

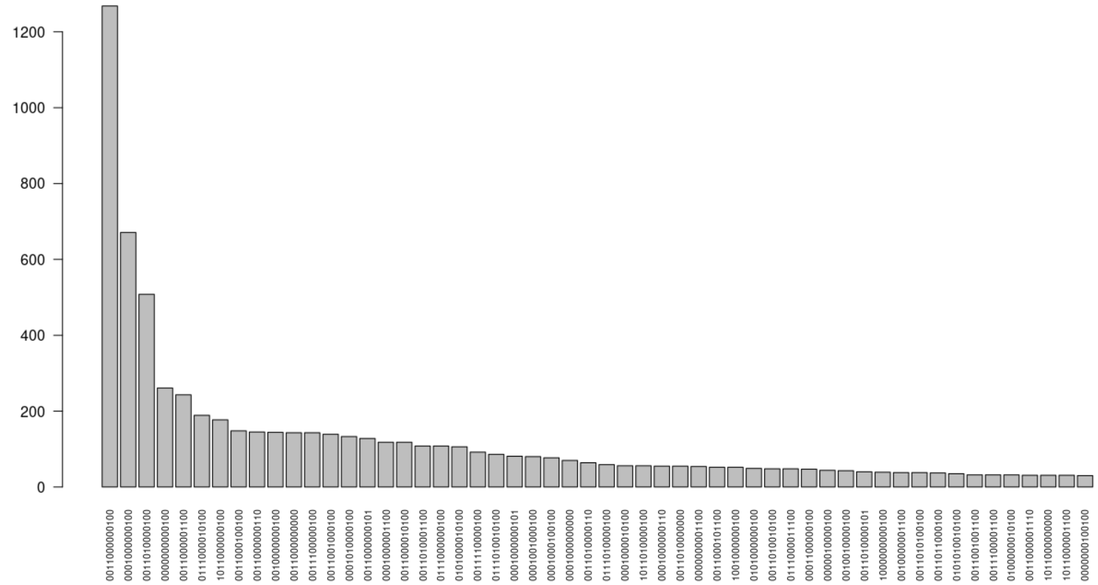

Figure S5: The reached states and their frequencies were determined after initializing the *lac* operon network from random initial states (based on a uniform distribution, which is the default option in `pastboon`) and performing 10,000 repeats using the BNp method, with all parameter values set to 0.1 and an asynchronous update scheme. Frequencies below 30 were ignored.

network has a unique steady-state distribution. The frequency of the reached states are obtained as follows:

```
reached_states <- get_reached_states(lac_operon_net_glucose,
    method = "BNp", params = rep(0.1, length(lac_operon_net_glucose$genes)),
    steps = 10, repeats = 10000, asynchronous = F)

reached_states_str <- apply(reached_states, 1, paste, collapse="")

freq <- sort(table(reached_states_str), decreasing = T)

barplot(freq[freq>=30], las=2, cex.names=.65)
```

Figure S5 shows resulting plot where the horizontal axis represents binary form of the reached states (based on the top-down order of the variables in Table S4) and the vertical axis shows frequencies (the frequencies below 30 were ignored). As Fig. S5 shows, the most frequent reached state is 0011000000100 which is equivalent of activation of R, Rm, and, Ge. The variables R and Rm represent concentration of locI that inhibits the expression of genes that encode proteins involved in the metabolism of lactose.

Next, we are going to study the reachability of state from each other in the condition of lacking glucose and the availability of a medium concentration of lactose. Thus, first we set $Ge_t = \text{off}$, $Le_t = \text{off}$, and $Lem_t = \text{on}$:

```
input_idx <- c(which(lac_operon_net$genes == "Ge"),
    which(lac_operon_net$genes == "Le"),
    which(lac_operon_net$genes == "Lem"))
```

```
lac_operon_net_lactose_medium <- fixGenes(lac_operon_net, input_idx, c(0, 0, 1))
```

In the next step, we generate a set of random states (considering just internal nodes):

```
states <- matrix(sample(c(0, 1), 10 * length(lac_operon_net_lactose_medium$genes),
    replace = T), nrow = 10, ncol = length(lac_operon_net_lactose_medium$genes))

colnames(states) <- lac_operon_net_lactose_medium$genes
rownames(states) <- apply(states, 1, paste, collapse="")

states[,"Ge"] <- 0
states[,"Le"] <- 0
states[,"Lem"] <- 1
```

To estimate the reachability of the states to each other, we use the function `count_pairwise_trans` which counts the number of direct or indirect transitions between a given set of states:

```
pairwise_trans <- count_pairwise_trans(lac_operon_net_lactose_medium,
    method = "BNp", params = rep(0.1, length(lac_operon_net_lactose_medium$genes)),
    states = states, steps = 10, repeats = 10000, asynchronous = F)

heatmap(pairwise_trans, labRow = rownames(states), labCol = rownames(states),
    Colv=NA, Rowv=NA, cexRow = 0.6, cexCol = 0.6)
```

Upon running the above code, the resulting pairwise transition frequency matrix is visualized as a heat-map as shown in Fig. S6.

In the next step, we are going to study the effect of altering parameter values over the steady-state activity of the nodes using the SDDS method. As described in the main text and also in Section S3.3, in `pastboon`, we implemented a generalization of the SDDS method in the case of binary variables (Boolean logic). Four parameters are associated with each node i: $\rho^{(i)}_{\mathsf{off}\to\mathsf{off}}$, $\rho^{(i)}_{\mathsf{off}\to\mathsf{on}}$, $\rho^{(i)}_{\mathsf{on}\to\mathsf{off}}$, and $\rho^{(i)}_{\mathsf{on}\to\mathsf{on}}$. To estimate the time-step at which the steady-state distribution of the network is reached, we use the `calc_node_activities` function to obtain activity time-series for all nodes, with all parameter values set to 0.9 (to ensure ergodicity, i.e., make the network independent of the initial node activity profiles) and using 50,000 repeats, 20 time-steps, and synchronous updating scheme:

```
p00 <- p01 <- p10 <- p11 <- rep(0.9,length(lac_operon_net$genes))

params <- list(p00 = p00, p01 = p01, p10 = p10, p11 = p11)

act <- calc_node_activities(lac_operon_net, method = "SDDS",
    params = params, steps = 20, repeats = 50000, asynchronous = F)


plot_node_activities(act, xlab = "Time-step", ylab = "Node activity")
```

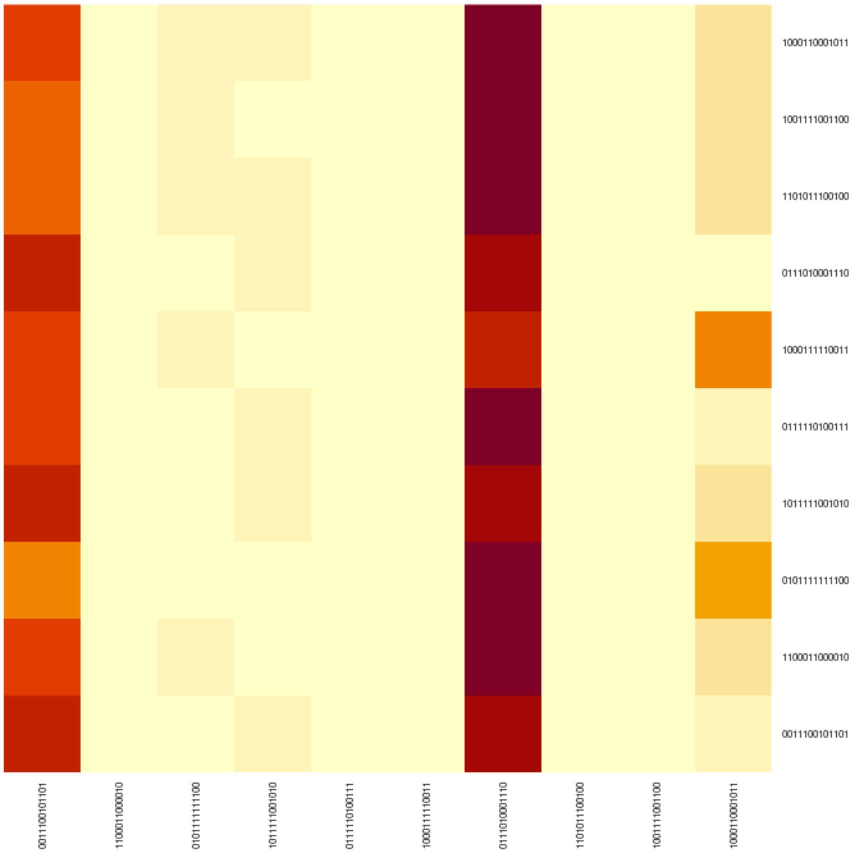

Figure S6: Pairwise transitions between a random set of states in the form of a heat-map. Each cell i, j shows how much state i reaches state j.

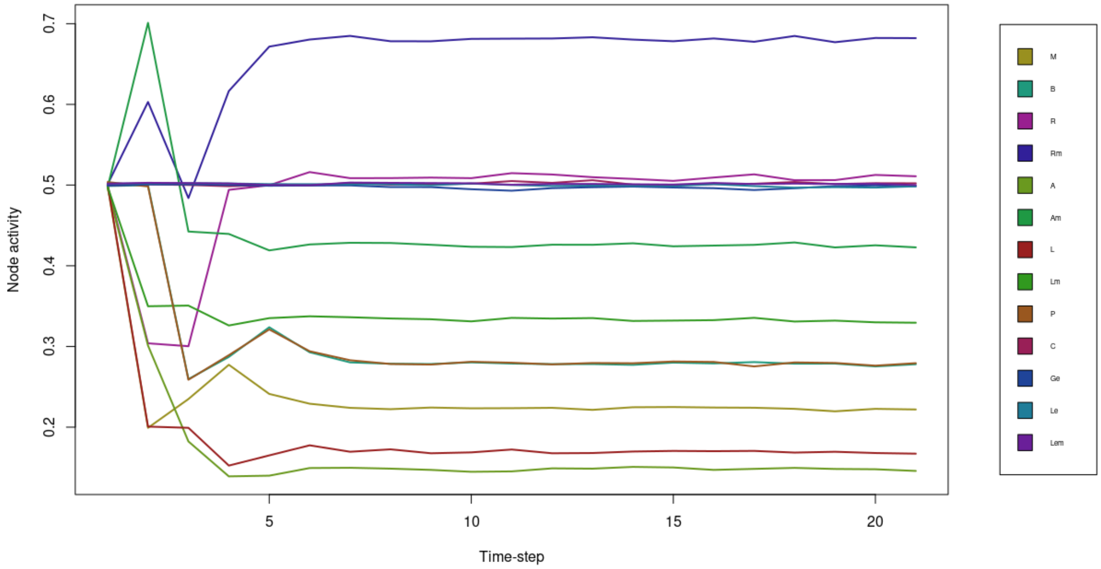

Figure S7: Time-series of activity of the *lac* operon network nodes for 20 time-steps and 50,000 repeats resulted from the `calc_node_activities` function with the SDDS method where all parameters were set to 0.9 and synchronous update is used.

Figure S7 shows the resulting plot. By visual inspection, we take into account the time-step 10 as the time-step at which the steady-state distribution is reached. To observe how lactose concentration affects *lacZ* mRNA concentration in the absence of glucose, we set the update functions of the nodes Ge, Le, and Lem as: $\mathit{Ge}_t = \mathsf{off}, \quad \mathit{Le}_t = \mathsf{on}, \quad \mathit{Lem}_t = \mathsf{on}$:

```
lac_operon_net_lactose <- fixGenes(lac_operon_net, input_idx, c(0, 1, 1))
```

The initial probabilities are set to 0 for the input nodes and 0.5 for the other nodes. It makes $\rho^{(Ge)}_{\mathsf{off}\to\mathsf{off}}$, $\rho^{(Le)}_{\mathsf{off}\to\mathsf{on}}$, $\rho^{(Le)}_{\mathsf{on}\to\mathsf{on}}$, $\rho^{(Lem)}_{\mathsf{off}\to\mathsf{on}}$, and $\rho^{(Lem)}_{\mathsf{on}\to\mathsf{on}}$ the only parameters influential over the input nodes:

```
initial_prob <- rep(0.5, length(lac_operon_net_lactose$genes))
initial_prob[input_idx] <- 0
```

We also set the parameter values to 0.9 for all nodes (except Ge, Le, and Lem). To ensure that the glucose concentration remains zero, we set $\rho^{(Ge)}_{\mathsf{off}\to\mathsf{off}} = 1$. Moreover, to have only two degrees of freedom (for visualization purposes), we set $\rho^{(Le)}_{\mathsf{off}\to\mathsf{on}} = \rho^{(Lem)}_{\mathsf{off}\to\mathsf{on}} = 1$ so that $\rho^{(Le)}_{\mathsf{on}\to\mathsf{on}}$ and $\rho^{(Lem)}_{\mathsf{on}\to\mathsf{on}}$ remain as adjustable parameters. For these two parameters, we chose values in the range $[0, 1]$ with an interval of 0.1. Overally, we set the parameter values according to Table 2 in the main text. The values of the parameters that do not have any effect on the input nodes are denoted by asterisks, which means that they can have any arbitrary value. The following code stores the steady-state activity (in the time-step $t = 10$ using 50,000 repeats) of node M (*lacZ* mRNA) as a proxy for the *lac* operon activity, resulting from various values of $\rho^{(Le)}_{\mathsf{on}\to\mathsf{on}}$ and $\rho^{(Lem)}_{\mathsf{on}\to\mathsf{on}}$:

```
values <- data.frame(L_e = numeric(), L_em = numeric(), M = numeric())
    for (L_e in seq(0,1,0.1)) {
      for (L_em in seq(0,1,0.1)) {
```

```
    params <- list(p00 = p00, p01 = p01, p10 = p10, p11 = p11)

    params$p00[11] <- 1
    params$p00[12] <- runif(1)
    params$p00[13] <- runif(1)

    params$p01[11] <- runif(1)
    params$p01[12] <- 1
    params$p01[13] <- 1

    params$p10[11] <- runif(1)
    params$p10[12] <- runif(1)
    params$p10[13] <- runif(1)

    params$p11[11] <- runif(1)
    params$p11[12] <- L_e
    params$p11[13] <- L_em

    nodeact <- calc_node_activities(lac_operon_net_lactose,
        method = "SDDS", params = params, initial_prob = initial_prob,
        steps = 10, repeats = 50000, last_step = T, asynchronous = F)

    values[nrow(values) + 1, ] <- c(L_e, L_em, nodeact['M'][[1]])
  }
}
```

The command `runif(1)` generates a random number in the range $[0, 1]$. Random values are assigned to some parameters to show their ineffectiveness (asterisks in Table 2 of the main text). The following code visualizes the results as a contour plot using the `plotly` package [12]:

```
L_e_unique <- sort(unique(values$L_e))
L_em_unique <- sort(unique(values$L_em))
M_matrix <- matrix(values$M, nrow = length(L_e_unique),
    ncol = length(L_em_unique), byrow = TRUE)

p <- plot_ly(x = ~L_e_unique, y = ~L_em_unique, z = ~M_matrix,
    type = "contour", colors = colorRamp(c("deepskyblue", "yellow",
    "red")), line = list(width = 1, color = "black"), colorbar =
    list(title="<i>M</i> (<i>lacZ</i> mRNA)"))

p <- p %>%
add_polygons(
  x = c(0, 0.99, 0, 0),
  y = c(0.01, 1, 1, 0.01),
  line = list(color = 'rgba(255,255,255,0.2)'),
```

```
  fillcolor = "rgba(255,255,255,0.6)",
  inherit = FALSE, showlegend = FALSE
)

ann = list(text = "Infeasible",  x = 0.3, y = 0.7,
    font=list(size=40,color="gray17"),textangle = -45, showarrow=FALSE)

xaxis_title <- TeX("p^{(Lem)}_{on→on}")
yaxis_title <- TeX("p^{(Le)}_{on→on}")

p <- p %>% layout(
    annotations = ann,
    xaxis = list(title = list(text = xaxis_title, standoff = 0,
        font = list(size = 100))),
    yaxis = list(title = list(text = yaxis_title, standoff = 0,
        font = list(size = 100)))
)

p

config(.Last.value, mathjax = "cdn")
```

The steady-state activity of node M for different values of $\rho^{(Le)}_{\text{on}\to\text{on}}$ and $\rho^{(Lem)}_{\text{on}\to\text{on}}$ is illustrated as a contour plot in Fig. S8. This plot demonstrates that the sensitivity of *lacZ* mRNA to Le is significantly greater than its sensitivity to Lem.

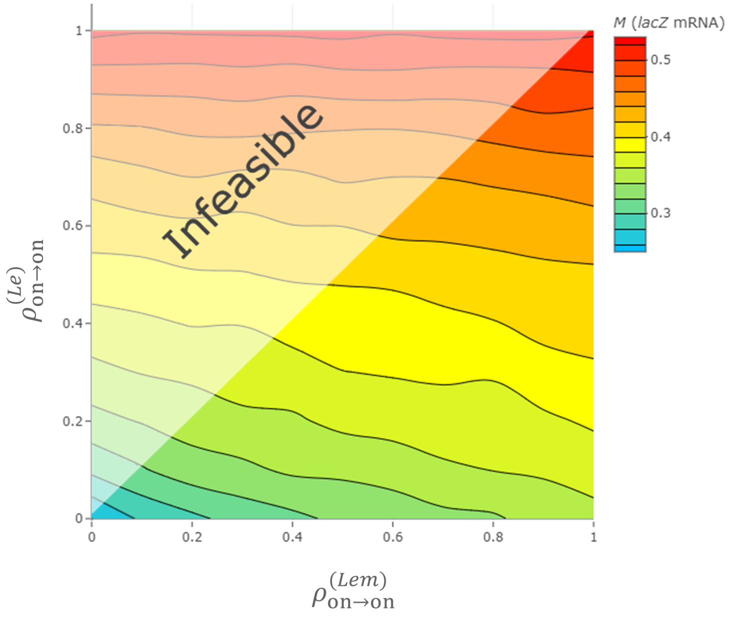

Figure S8: Effect of Le and Lem on M (*lacZ* mRNA) using the SDDS method.

Finally, here we show how the PEW method can be used to study the effect of perturbing an edge over steady-state activity of the variables. As mentioned in the main text, when there is no fixed node (a node without any input edge) by setting the p_{on} and p_{off} parameters of all edges to the values in the range $(0, 1)$, ergodicity of the network is guaranteed. Meanwhile, in the case of having fixed nodes, as is the case with the *lac* operon network, if the p_{on} and p_{off} parameters of all edges are set to the values in the range $(0, 1)$ the ergodicity of the "subnetwork of internal nodes" is guaranteed. Here, we obtain the activity time-series of the internal nodes of the *lac* operon BN based on the PEW method when all parameters are set to 0.9 and the input nodes are set as *Ge*=off, *Le*=off, and, *Lem*=on (to emulate the lack of glucose and availability of medium concentration of lactose):

```
edges <- extract_edges(lac_operon_net_lactose_medium)

p_on <- rep(0.9, nrow(edges))
p_off <- rep(0.9, nrow(edges))

params <- list(p_on = p_on, p_off = p_off)

node_act <- calc_node_activities(lac_operon_net_lactose_medium, method = "PEW",
    params = params, steps = 20, repeats = 50000, asynchronous = F)

plot_node_activities(node_act, xlab = "Time-step", ylab = "Node activity")
```

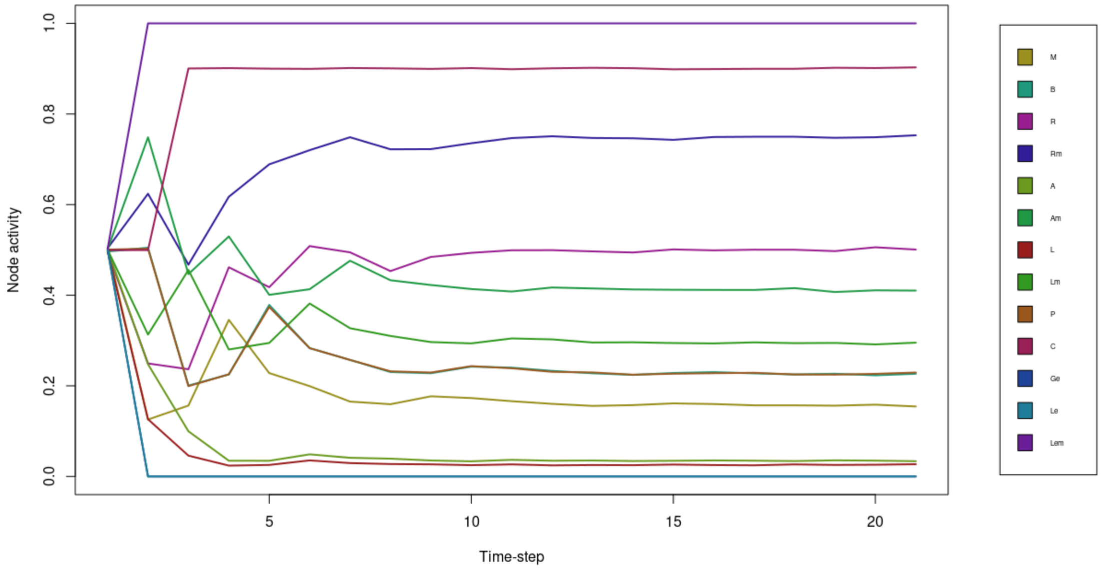

Figure S9: Time-series of the activity of *lac* operon network nodes for 20 time-steps and 50,000 repeats resulted from the `calc_node_activities` function with the PEW method where all parameters were set to 0.9 and synchronous update is used.

Figure S9 shows the resulting time-series of the nodes. By visual inspection, we take into account the time-step 10 as the time-step at which the steady-state distribution is reached. We choose the

edge with the source B and destination A to perturb. The values of the parameters $p_{\mathsf{on}}^{\mathsf{R}}$ and $p_{\mathsf{off}}^{\mathsf{A\text{-}R}}$ are chosen such that they are summed up to 1 (dotted line in Fig. S3):

```
p <- rep(0.9, length(lac_operon_net_lactose_medium$genes))

params <- list(p00 = p, p01 = p, p10 = p, p11 = p)

edge_idx <- which(edges$source == "A" & edges$destination == "R")

var_target <- "M"

values <- data.frame(matrix(ncol = length(lac_operon_net_lactose_medium$genes) + 2,
    nrow = 0))

colnames(values) <- c("p_on", "p_off", lac_operon_net_lactose_medium$genes)

values[] <- lapply(values, as.numeric)

for (v in seq(0, 1, 0.1)) {
    params <- list(p_on = p_on, p_off = p_off)

    params$p_on[edge_idx] <- v
    params$p_off[edge_idx] <- 1 - v

    nodeact <- calc_node_activities(lac_operon_net_lactose_medium,
        method = "PEW", params = params, steps = 10, repeats = 50000,
        last_step = T, asynchronous = F)

    values[nrow(values) + 1, ] <- c(v, 1 - v, nodeact)
}

plot_node_activities(values[,3:ncol(values)],
    xlab = expression(paste("p"[on]^"(A-R)", " ",
    "(", " = 1 - p"[off]^"(A-R)", ")")), ylab = "Node activity")
```

Figure S10 shows the resulting plot which is steady-state activity of the nodes for different values of $p_{\mathsf{on}}^{\mathsf{A\text{-}R}}$ and $p_{\mathsf{off}}^{\mathsf{A\text{-}R}}$.

S4.2 The example of myeloid differentiation BN

As another example, we use `pastboon` to analyze a Boolean model of myeloid differentiation gene regulatory network [11] which consists of 11 nodes. This network shows the differentiation mechanism of common myeloid progenitors into erythrocytes, megakaryocytes, granulocytes, and monocytes. The descriptions of the variables for the myeloid differentiation BN are shown in Table S5.

The update rules governing evolution of the myeloid differentation network are as follows:

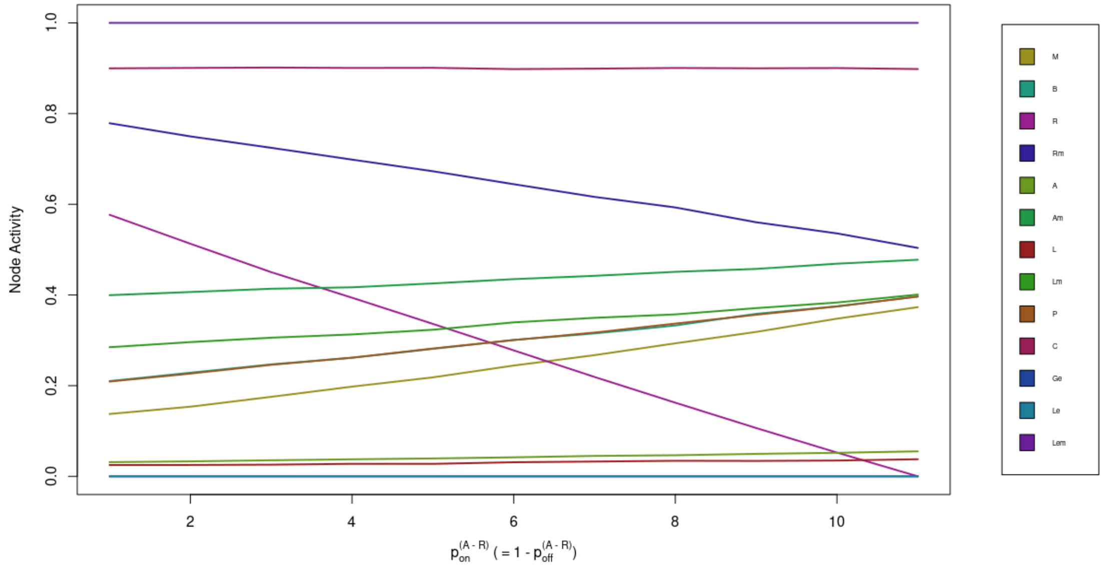

Figure S10: Steady-state activity of the *lac* operon network nodes for different values of the parameter $p_{\mathsf{on}}^{\mathsf{A\text{-}R}}$ $(= 1 - p_{\mathsf{off}}^{\mathsf{A\text{-}R}})$.

$$
\begin{aligned}
&\mathit{GATA2}_t = \mathit{GATA2}_{t-1} \wedge \neg\left(\mathit{GATA1}_{t-1} \wedge \mathit{FOG1}_{t-1}\right) \wedge \neg \mathit{PU1}_{t-1}\\
&\mathit{GATA1}_t = \left(\mathit{GATA1}_{t-1} \vee \mathit{GATA2}_{t-1} \vee \mathit{Fli1}_{t-1}\right) \wedge \neg \mathit{PU1}_{t-1}\\
&\mathit{FOG1}_t = \mathit{GATA1}_{t-1}\\
&\mathit{EKLF}_t = \mathit{GATA1}_{t-1} \wedge \neg \mathit{Fli1}_{t-1}\\
&\mathit{Fli1}_t = \mathit{GATA1}_{t-1} \wedge \neg \mathit{EKLF}_{t-1}\\
&\mathit{SCL}_t = \mathit{GATA1}_{t-1} \wedge \neg \mathit{PU1}_{t-1}\\
&\mathit{CEBPa}_t = \mathit{CEBPa}_{t-1} \wedge \neg\left(\mathit{GATA1}_{t-1} \wedge \mathit{FOG1}_{t-1} \wedge \mathit{SCL}_{t-1}\right)\\
&\mathit{PU1}_t = \left(\mathit{CEBP}\alpha_{t-1} \vee \mathit{PU1}_{t-1}\right) \wedge \neg\left(\mathit{GATA1}_{t-1} \vee \mathit{GATA2}_{t-1}\right)\\
&\mathit{cJun}_t = \mathit{PU1}_{t-1} \wedge \neg \mathit{Gfi1}_{t-1}\\
&\mathit{EgrNab}_t = \left(\mathit{PU1}_{t-1} \wedge \mathit{cJun}_{t-1}\right) \wedge \neg \mathit{Gfi1}_{t-1}\\
&\mathit{Gfi1}_t = \mathit{CEBP}\alpha_{t-1} \wedge \neg \mathit{EgrNab}_{t-1}
\end{aligned}
\tag{S3}
$$

Like the *lac* operon example, we set the random seed to the fixed value of 912 to be able to reproduce the results:

```
set.seed(912)
```

Also, we load the required packages `pastboon` and `plotly`:

```
library(pastboon)
library(BoolNet)
library(plotly)
```

Now we load the myeloid differentiation BN using the following command:

Table S5: The description of myeloid differentiation network variables.

Gene name	Variable name	Concentration of:
GATA-2	*GATA2*	Early MegE factor
GATA-1	*GATA1*	Central MegE factor
FOG-1	*FOG1*	GATA-1 cofactor
EKLF	*EKLF*	Erythroid factor
Fli-1	*Fli1*	Megakaryocytic factor
SCL	*SCL*	Central hematopoietic player, involved in MegE differentiation
C/EBPα	*CEBPa*	Early GM factor
PU1	*PU1*	Central GM factor
cJun	*cJun*	The Jun transcription factor
EgrNab	*EgrNab*	Integrated monocytic factor
Gfi-1	*Gfi1*	Granulocytic factor

```
data(myeloid_diff_net)
```

Moreover, to plot activity of the nodes, we first generate a set of distinct colors (equal to the number of variables):

```
generate_colors <- function(n) {
    hues <- seq(0, 1, length.out = n + 1)[-1]
    s <- 0.8
    v <- 0.6
    colors <- hsv(h = hues, s = s, v = v)
    return(colors)
}

col_vec <- generate_colors(length(myeloid_diff_net$genes))
col_vec <- col_vec[sample(1:length(col_vec))]
```

Then, we define the following function for the purpose of visualizing activity time-series:

```
plot_node_activities <- function(node_activities, xlab, ylab) {
  old_par <- par(no.readonly = TRUE)
  layout(matrix(c(1, 2), nrow = 1), width = c(4, 1))
  par(mar = c(5, 4, 4, 0))
  matplot(1:nrow(node_activities), node_activities, type = "l",
      frame = TRUE, lwd = 2, lty = 1, xlab = xlab, ylab = ylab,
      col = col_vec)
  par(mar = c(5, 0, 4, 2))
  plot(c(0, 1), type = "n", axes = FALSE, xlab = "")
```

```
  legend("center", colnames(node_activities), col = col_vec,
         cex = 0.5, fill = col_vec)
  layout(matrix(1))
  par(old_par)
}
```

Starting from an early hematopoietic state where only the nodes *CEBPa*, *PU1*, and *GATA2* are active (on) and the rest of nodes are inactive (off) and using an asynchronous updating scheme, four attractor states are reached. Because of the stochastic nature of asynchronous update, at each simulation, by starting from the same initial state, a different attractor is reached. Figure S11 shows the activation patterns for the four attractors which were obtained using the `getAttractors` function of the `BoolNet` package in `R`.

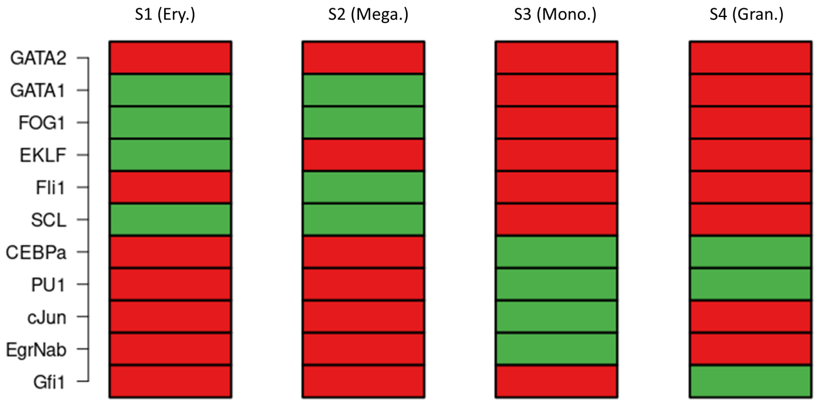

Figure S11: The attractors of the myeloid differentiation network by starting from an early hematopoietic state where only the nodes *CEBPa*, *PU1*, and *GATA2* are active (on) and the rest of nodes are inactive (off) and using an asynchronous updating scheme. The attractors S1, S2, S3, and S4 represent the differentiated cells erythrocytes, megakaryocytes, monocytes, and granulocytes, respectively.

According to Fig. S11, for each attractor (representing a cell-type) we can define a proxy variable that shows the system is in that attractor state when a stable state is reached. More precisely, for the attractors S1, S2, S3, and S4, one can take into account the variables *EKLF*, *Fli1*, *cJun*, and *Gfi1*, as their corresponding proxy, respectively.

We first use the BNp method to obtain the activity time-series of the nodes for 300 time-steps using asynchronous update and 50,000 repeats. To guarantee ergodicity of the network (avoiding getting stuck in an attractor state), we set all parameter values to 0.1 (refer to the main text for description of the BNp method). The following code produces and plots the activity time-series of the nodes using the mentioned parameter setting:

```
act <- calc_node_activities(myeloid_diff_net, method = "BNp",
    params = rep(0.1, length(myeloid_diff_net$genes)), steps = 300,
    repeats = 50000)

plot_node_activities(act, xlab = "Time-step", ylab = "Node activity")
```

After running the above code, the activity rate of the nodes for 50,000 repeats and 300 time-steps using asynchronous update is calculated and stored in the `act` variable and visualized as presented

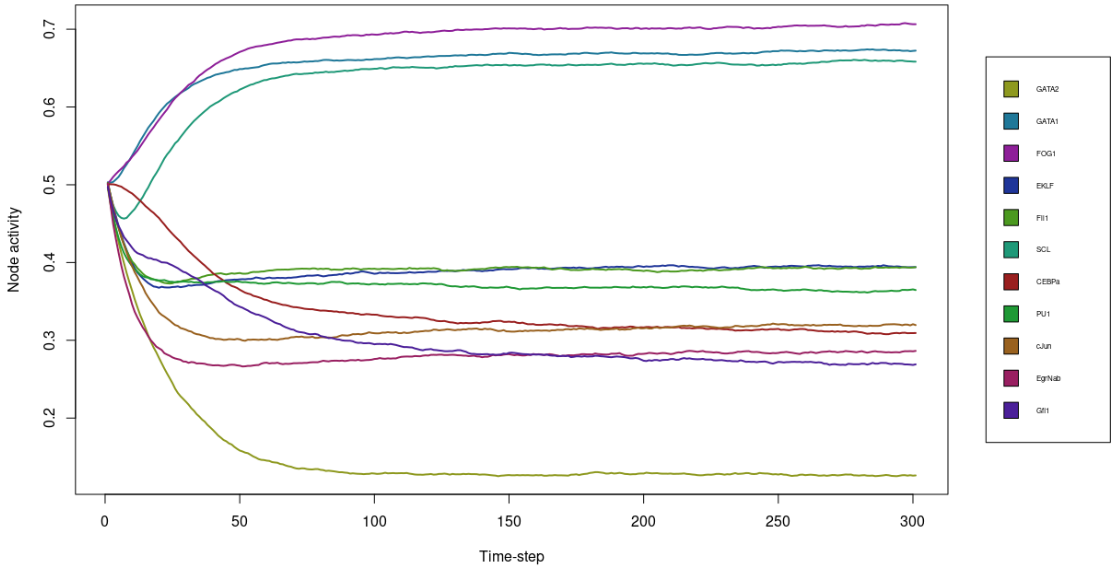

Figure S12: Time-series of the activity of myeloid differentiation network nodes for 300 time-steps and 50,000 repeats resulted from the `calc_node_activities` function with the BNp method where all parameters were set to 0.1 and asynchronous update is used.

in Fig. S12. Please note that since the updating scheme used in the main article of the myeloid differentiation network [11] is asynchronous update, here asynchronous update is used which is the default update method in the `pastboon` package. Now, we can estimate steady-state time-step using the following command:

```
calc_convergence_time(act, 0.001, window_size = 5)
```

The above command returns the time-step at which the changes in all curves are below 0.001 for 5 consecutive time-steps. Here, the function returns 167. Now, we define the initial hematopoietic state as follows:

```
initial_state <- rep(0, length(myeloid_diff_net$genes))

active_nodes <- c("CEBPa","PU1", "GATA2")

initial_state[myeloid_diff_net$genes %in% active_nodes] <- 1
```

To study the probability of reaching each attractor state from the initial hematopoietic state, here we use the package's `get_reached_states` function to observe which states are most frequently reached after 167 time-steps (steady-state) using 10,000 repeats. The rest of the arguments are set to the same value as the setting of `calc_node_activities` function. Since all the parameters related to the BNp method are set to 0.1, the network is prevented from getting stuck in an attractor state. The frequency of the reached states are obtained as follows:

```
reached_states <- get_reached_states(myeloid_diff_net,
    method = "BNp", params = rep(0.1, length(myeloid_diff_net$genes)),
```

```
    initial_states = initial_state, steps = 167, repeats = 10000)

reached_states_str <- apply(reached_states, 1, paste, collapse="")

freq <- sort(table(reached_states_str), decreasing = T)

barplot(freq[freq >= 15], las=2, cex.names=.65)
```

Using the above commands, the number of times that each state is reached from the other states after 167 time-steps is visualized in the form of a bar-plot which is presented in Fig. S13. The horizontal axis represents the binary form of the reached states (based on the top-down order of the variables in Table S5) and the vertical axis shows frequencies (the frequencies below 15 were ignored). As the figure shows, the first four most frequent reached states are 01110100000 (S1), 01101100000 (S2), 00000011001 (S4), and, 00000011110 (S3). Also, from the figure it is turned out that the probability that a myeloid progenitor cell is differentiated into the S1 and S2 cell-types (erythrocyte and megakaryocyte) is greater than that of the others with our parameter setting.

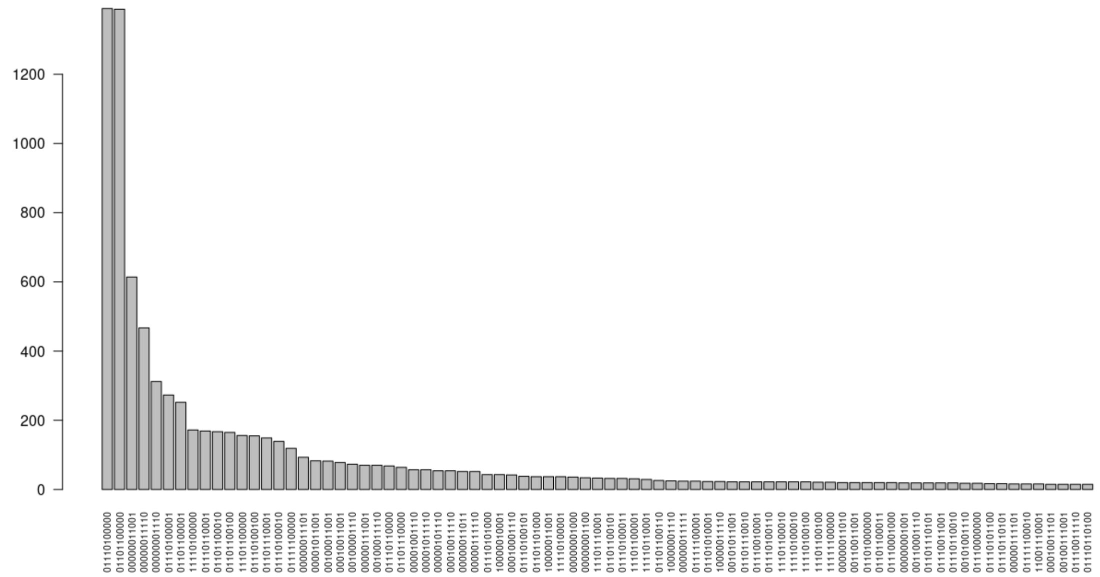

Figure S13: The reached states and their frequencies after starting the myeloid differentiation network from the initial hematopoietic state and 10,000 repeats when using the BNp method, with all parameter values set to 0.1 and an asynchronous update scheme. Frequencies below 15 were ignored.

Now, we are going to see how much the four states corresponding to the differentiated cell-types are reachable from each other. To do so, we use the function `count_pairwise_trans` which counts the number of direct or indirect transitions between a given set of states.

```
states <- rbind(c(0, 1, 1, 1, 0, 1, 0, 0, 0, 0, 0), # S1
                c(0, 1, 1, 0, 1, 1, 0, 0, 0, 0, 0), # S2
                c(0, 0, 0, 0, 0, 0, 1, 1, 1, 1, 0), # S3
                c(0, 0, 0, 0, 0, 0, 1, 1, 0, 0, 1)) # S4
```

```
rownames(states) <- c("S1", "S2", "S3", "S4")

pairwise_trans <- count_pairwise_trans(myeloid_diff_net,
    method = "BNp", params = rep(0.1, length(myeloid_diff_net$genes)),
    states = states, steps = 167, repeats = 10000)

heatmap(pairwise_trans, labRow =  rownames(states), labCol = rownames(states),
        cexRow = 0.85, cexCol = 0.85)
```

The above code produces a heat-map showing the number of transitions between the four states corresponding to the differentiated cell-types as represented in Fig. S14. The cluster structure of the states approves hierarchical decision-making in differentiation of common myeloid progenitors, first into megakaryocyte-erythrocyte and granulocyte-monocyte progenitors, and then megakaryocyte-erythrocyte into erythrocyte (S1), erythrocyte (S2), and granulocyte-monocyte into monocyte (S3), granulocyte (S4).

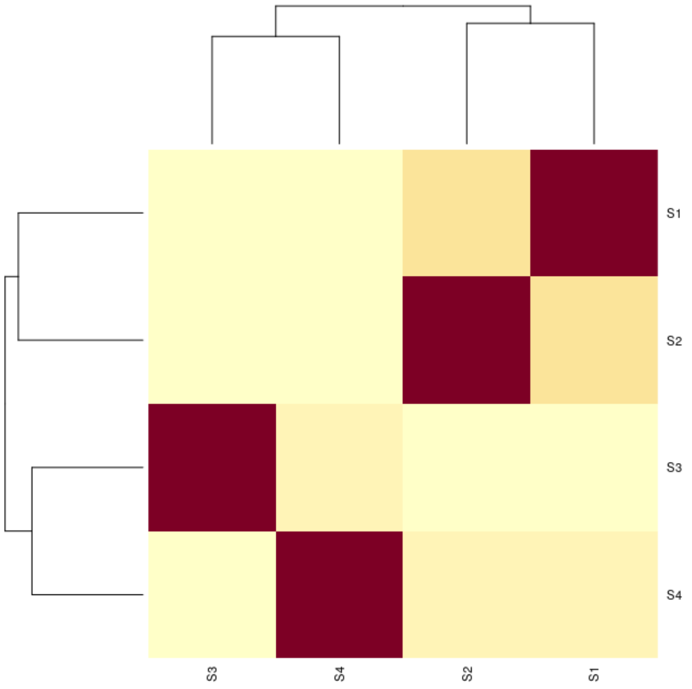

Figure S14: Pairwise transitions between the four states corresponding to the differentiated cell-types in the form of a heat-map. Each cell i, j shows how much state i reaches state j.

In the next step, we study the effect of the variables on each other in steady-state distribution. To do so, we use the SDDS method which enables us to control the tendency of variables to be active (on) or inactive (off). First we need to estimate the time-step at which the steady-state distribution of the network is reached. Based on the SDDS model, we set all parameter values of all nodes to 0.9. Then using the following code we obtain the time-series representing node activities in consecutive time-steps:

```
p <- rep(0.9, length(myeloid_diff_net$genes))
```

```
params <- list(p00 = p, p01 = p, p10 = p, p11 = p)

act <- calc_node_activities(myeloid_diff_net,
    method = "SDDS", params = params, steps = 300, repeats = 50000)

plot_node_activities(act, xlab = "Time-step", ylab = "Node activity")
```

Using the `plot_node_activities` function we defined before (refer to Section S4.1), the activity time-series of the nodes is visualized. Figure S15 shows the resulting plot where each curve represents the activity of a distinct variable in different time-steps. Since all parameters of the SDDS method are set to 0.9, it is guaranteed that the corresponding Markov chain of the network has a unique steady-state distribution. This means that the activity pattern of the nodes is independent of the initial state of the nodes. By visual inspection, we take into account the time-step 200 as the time-step at which the steady-state distribution is reached.

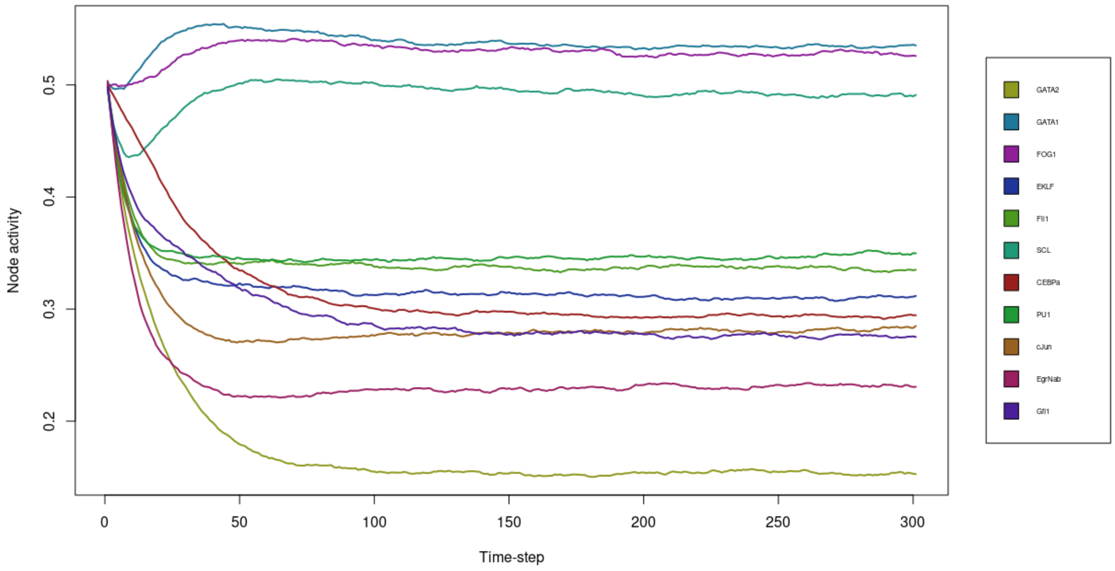

Figure S15: Time-series of the activity of myeloid differentiation network nodes for 300 time-steps and 50,000 repeats resulted from the `calc_node_activities` function with the SDDS method where all parameters were set to 0.9 and asynchronous update is used.

By having an estimation from the steady-state time-step (200 in our case), we can study the effect of altering parameter values over the steady-state activity of the nodes. Thus, for each of the four proxy nodes, we manually select two upstream nodes to study their effect on the proxy node. To do so, we fix the two selected upstream variables to on. Fixing a node i to on makes the parameters $\rho^{(i)}_{\mathsf{off}\to\mathsf{off}}$ and $\rho^{(i)}_{\mathsf{on}\to\mathsf{off}}$ useless because the node i is never updated to off (see Section S1). Thus, only the parameters $\rho^{(i)}_{\mathsf{off}\to\mathsf{on}}$ and $\rho^{(i)}_{\mathsf{on}\to\mathsf{on}}$ affect node state. We use the following code to store node activities for different parameter values:

```
p <- rep(0.9, length(myeloid_diff_net$genes))
params <- list(p00 = p, p01 = p, p10 = p, p11 = p)
```

```
var1 <- "PU1"
var2 <- "Fli1"
var3 <- "EKLF"

var1_idx <- which(myeloid_diff_net$genes == var1)
var2_idx <- which(myeloid_diff_net$genes == var2)

myeloid_diff_net_fixed <- fixGenes(myeloid_diff_net,
    c(var1_idx, var2_idx), 1)

values <- data.frame(var1 = numeric(), var2 = numeric(), var3 = numeric())

for (v1 in seq(0,1,0.1)) {
  for (v2 in seq(0,1,0.1)) {

    params <- list(p00 = p, p01 = p, p10 = p, p11 = p)

    params$p00[var1_idx] <- runif(1)
    params$p00[var2_idx] <- runif(1)
    params$p01[var1_idx] <- 0.9
    params$p01[var2_idx] <- 0.9
    params$p10[var1_idx] <- runif(1)
    params$p10[var2_idx] <- runif(1)
    params$p11[var1_idx] <- v1
    params$p11[var2_idx] <- v2

    nodeact <- calc_node_activities(myeloid_diff_net_fixed,
        method = "SDDS", params = params, initial_prob = initial_state,
        steps = 200, repeats = 50000, last_step = T)

    values[nrow(values) + 1, ] <- c(v1, v2, nodeact[var3][[1]])
  }
}
```

While the parameters of the other nodes are set to 0.9, we store the steady-state activity of the target proxy node for different parameter values of its two upstream nodes. We can use the following commands to visualize the results as a contour plot using the `plotly` package [12]:

```
var1_unique <- sort(unique(values$var1))
var2_unique <- sort(unique(values$var2))
var3_matrix <- matrix(values$var3, nrow = length(var1_unique),
    ncol = length(var2_unique), byrow = TRUE)

p <- plot_ly(x = ~var1_unique, y = ~var2_unique, z = ~var3_matrix,
```

```
    type = "contour", colors = colorRamp(c("deepskyblue", "yellow", "red")),
    line = list(width = 1, color = "black"), colorbar=list(title=var3))

xaxis_title <- TeX(paste0("p^{(", var2, ")}_{on→on}"))
yaxis_title <- TeX(paste0("p^{(", var1, ")}_{on→on}"))

p <- p %>% layout(
  xaxis = list(title = list(text = xaxis_title, standoff = 0,
      font = list(size = 100))),
  yaxis = list(title = list(text = yaxis_title, standoff = 0,
      font = list(size = 100)))
)

p

config(.Last.value, mathjax = 'cdn')
```

Figure S16 shows the resulting plot for each of the proxy variables. Finally, we are going to show how the PEW method can be used to study the effect of perturbing an edge over steady-state activity of the variables. As mentioned in the main text, when there is no fixed node (a node without any input edge) by setting the parameters p_{on} and p_{off} of all edges to values in the range $(0, 1)$, ergodicity of the network is guaranteed.

```
edges <- extract_edges(myeloid_diff_net)

p_on <- rep(0.9, nrow(edges))
p_off <- rep(0.9, nrow(edges))
params <- list(p_on = p_on, p_off = p_off)

act <- calc_node_activities(myeloid_diff_net,
  method = "PEW", params = params, steps = 300, repeats = 50000)

plot_node_activities(act, xlab = "Time-step", ylab = "Node activity")
```

Figure S17 shows the activity time-series of the nodes based on the PEW method when all parameters are set to 0.9. By visual inspection, we take into account the time-step 200 as the steady-state time-step. We also choose the edge with the source *GATA1* and destination *PU1* to perturb. The values of the parameters $p_{\text{on}}^{\text{GATA1-PU1}}$ and $p_{\text{off}}^{\text{GATA1-PU1}}$ are chosen such that they are summed up to 1 (dotted line in Fig. S3). We are going to study the effect of perturbing the edge GATA1-PU1 over steady-state activity of the EKLF variable:

```
p <- rep(0.9, length(myeloid_diff_net$genes))

params <- list(p00 = p, p01 = p, p10 = p, p11 = p)
```

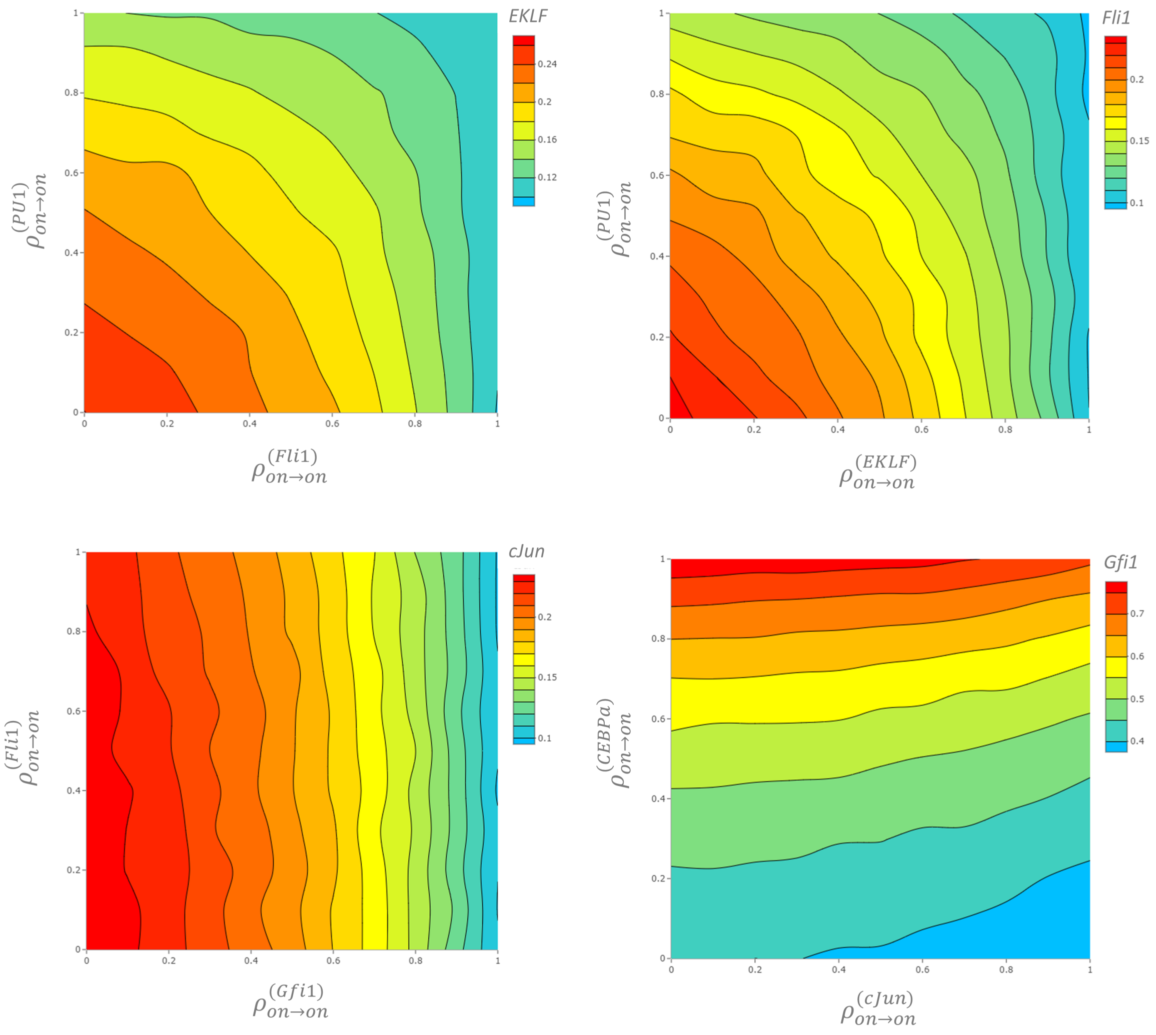

Figure S16: Effect of two manually selected upstream nodes over the target nodes representing attractors of the network.

```
edge_idx <- which(edges$source == "GATA1" & edges$destination == "PU1")
var_target <- "EKLF"

values <- data.frame(matrix(ncol = length(p) + 2, nrow = 0))
colnames(values) <- c("p_on", "p_off", myeloid_diff_net$genes)
values[] <- lapply(values, as.numeric)

for (v in seq(0,1,0.1)) {
  params <- list(p_on = p_on, p_off = p_off)

  params$p_on[edge_idx] <- v
  params$p_off[edge_idx] <- 1 - v

  nodeact <- calc_node_activities(myeloid_diff_net,
```

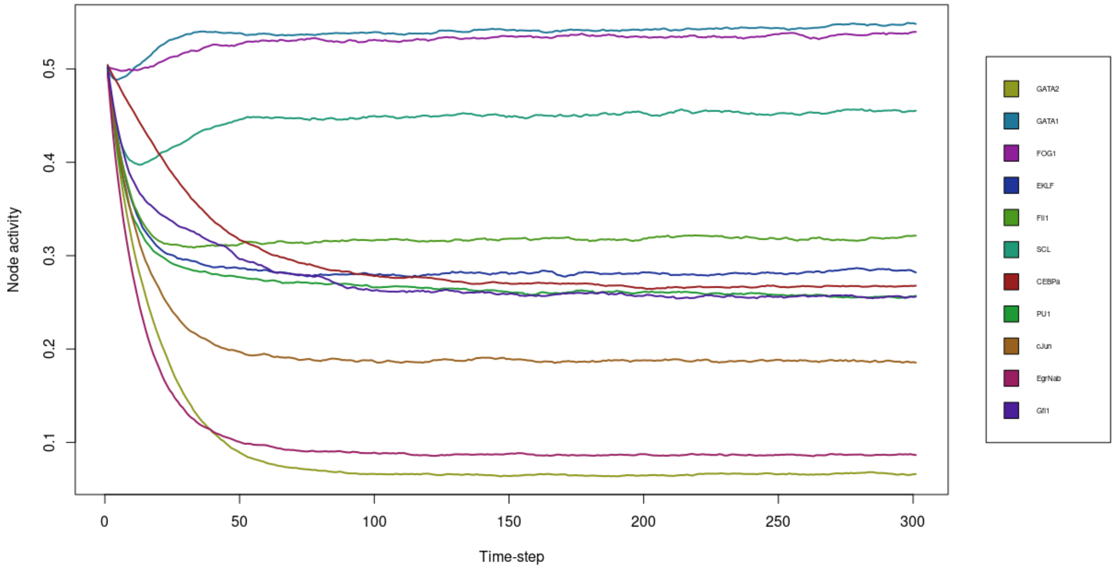

Figure S17: Time-series of the activity of myeloid differentiation network nodes for 300 time-steps and 50,000 repeats resulted from the `calc_node_activities` function with the PEW method where all parameters were set to 0.9 and asynchronous update is used.

```
        method = "PEW", params = params, initial_prob = initial_state,
        steps = 200, repeats = 50000, last_step = T)

    values[nrow(values) + 1, ] <- c(v, 1 - v, nodeact)
  }

  plot_node_activities(values[,3:ncol(values)],
      xlab = expression(paste("p"[on]^"(GATA1-PU1)", " ",
      "(", " = 1 - p"[off]^"(GATA1-PU1)", ")")), ylab = "Node Activity")
```

Figure S18 shows the resulting plot which is steady-state activity of the nodes for different values of $p_{\text{on}}^{\text{GATA1-PU1}}$ and $p_{\text{off}}^{\text{GATA1-PU1}}$.

S5 Future developments

In the initial implementation of `pastboon`, we prioritized including essential functions to enable researchers to model and analyze various complex systems effectively. These functions lay the foundation for simulating, visualizing, and interpreting the behavior of BNs. Future versions of the package will introduce more parameterization techniques to expand its versatility.

In addition, we plan to incorporate features such as parallelized simulations to enhance computational efficiency, improved visualization tools for better interpretation of results, and advanced methods for parameter estimation. These planned developments aim to make `pastboon` a comprehensive and scalable toolkit for researchers working on complex systems.

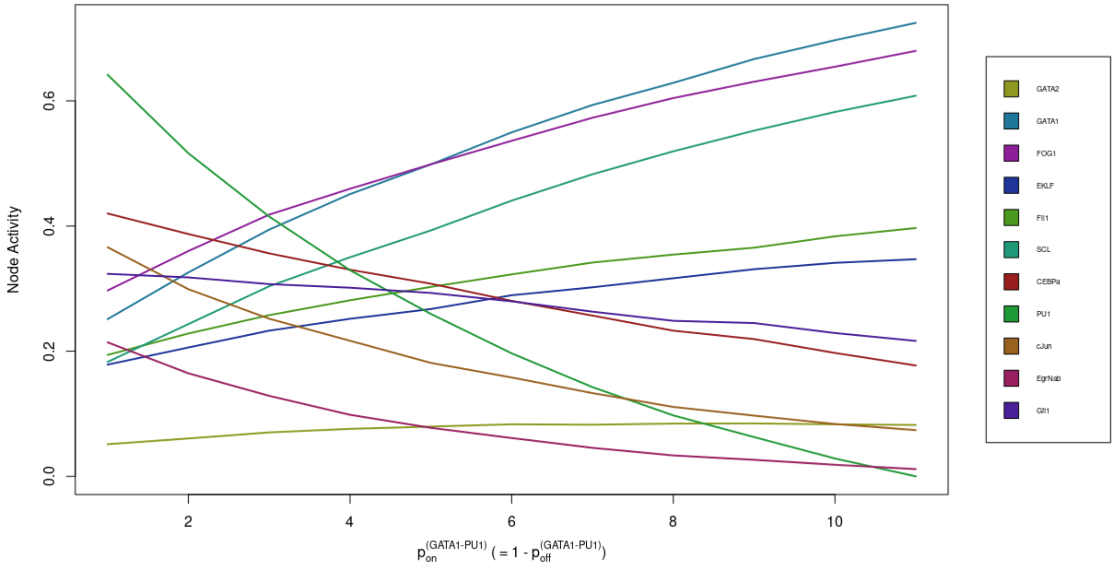

Figure S18: Steady-state activity of the myeloid differentiation network nodes for different values of the parameter $p_{\text{on}}^{\text{GATA1-PU1}}$ $(= 1 - p_{\text{off}}^{\text{GATA1-PU1}})$.

References

[1] Gautier Stoll, Eric Viara, Emmanuel Barillot, and Laurence Calzone. Continuous time boolean modeling for biological signaling: application of gillespie algorithm. *BMC systems biology*, 6:1–18, 2012.

[2] Gautier Stoll, Barthélémy Caron, Eric Viara, Aurélien Dugourd, Andrei Zinovyev, Aurélien Naldi, Guido Kroemer, Emmanuel Barillot, and Laurence Calzone. Maboss 2.0: an environment for stochastic boolean modeling. *Bioinformatics*, 33(14):2226–2228, 2017.

[3] Levi D Mcclenny, Mahdi Imani, and Ulisses M Braga-Neto. Boolfilter: an r package for estimation and identification of partially-observed boolean dynamical systems. *BMC bioinformatics*, 18:1–8, 2017.

[4] Tomáš Helikar, Brian Kowal, and Azamat Madrahimov. The cell collective: toward an open and collaborative approach to systems biology. *BMC Systems Biology*, 6(1):96, 2012.

[5] Christoph Müssel, Markus Hopfensitz, and Hans A Kestler. Boolnet—an r package for the generation, reconstruction, and analysis of boolean networks. *Bioinformatics*, 26(10):1378–1380, 2010.

[6] Réka Albert and Hans G Othmer. Boolean modeling of genetic regulatory networks. *Journal of Theoretical Biology*, 223(1):1–18, 2003.

[7] Anthony Szedlak, Giovanni Paternostro, and Carlo Piermarocchi. Control of asymmetric hopfield networks and application to cancer attractors. *PloS one*, 9(8):e105842, 2014.

[8] D Murrugarra, A Veliz-Cuba, B Aguilar, S Arat, and R Laubenbacher. Modeling stochasticity and variability in gene regulatory networks. *EURASIP J Bioinform Syst Biol*, 2012:5, 2012.

[9] D Deritei, N Kunšič, and P Csermely. Probabilistic edge weights fine-tune Boolean network dynamics. *PLoS Comput Biol*, 18:e1010536, 2022.

[10] A Veliz-Cuba and B Stigler. Boolean models can explain bistability in the *lac* operon. *J Comput Biol*, 18(6):783–794, 2011.

[11] Jan Krumsiek, Carsten Marr, Timm Schroeder, and Fabian J Theis. Hierarchical differentiation of myeloid progenitors is encoded in the transcription factor network. *PloS one*, 6(8):e22649, 2011.

[12] Carson Sievert. Interactive Web-Based data visualization with r, plotly, and shiny, 2020.
